# The effects of promoter variations of the N-Methylcanadine 1-Hydroxylase (CYP82Y1) gene on the noscapine production in opium poppy

**DOI:** 10.1038/s41598-018-23351-0

**Published:** 2018-03-21

**Authors:** Davar Abedini, Sajad Rashidi Monfared, Alireza Abbasi

**Affiliations:** 1 Department of Biotechnology, Faculty of Agriculture, Tarbiat Modares University, Tehran, Iran; 2 Department of Agronomy and Plant Breeding, University of Tehran, Karaj, Iran

## Abstract

Noscapine is an antitumor alkaloid produced in opium poppy (*Papaver somniferum*) and some members of the Papaveraceae family. It has been primarily used for its antitussive effects; more recently, its anticancer properties were shown. Herein, we detected an SSR embedded in the promoter region of the CYP82Y1 gene, which was found to be the first committed-step enzyme in the noscapine biosynthesis pathway, using the MISA program. Some collected ecotypes of *P. somniferum* were investigated for understanding of SSRs role in the regulation of gene expression and metabolite content. Quantitative PCR showed that a variation in the motif repeat number (either a decrease or increase) down-regulated the expression of the CYP82Y1 gene. Furthermore, the analysis of noscapine content suggested that a variation in the promoter region influence noscapine amount. Moreover, *P. bracteatum* was analyzed in both transcript and metabolite levels, and illustrated much less expression and metabolite level in comparison to *P. somniferum*. By exploiting the transcriptome data from the eight genera of the Papaveraceae family, we found that noscapine biosynthesis genes are present in *P. bracteatum* and are not shared in other genera of the Papaveraceae family. This results may explain production of a confined metabolite within a genus.

## Introduction

Opium poppy, *Papaver somniferum* L., a member of Papaveraceae family, is one of the most agronomically and economically important plant and is one of the ancient herbal plants with powerful pharmacological features^1,2^. Even though more than 40 alkaloids have been identified in poppy, six represent almost all of the total alkaloid including: morphine (4–21%), thebaine (0.5–2%), codeine (0.8–2.5%), noscapine (4–8%), papaverine (0.5–2.5%), and reticuline (0.1–2%)^1,3^. Noscapine belongs to benzylisoquinoline alkaloid produced in opium poppy and other members of the Papaveraceae family. Noscapine has been used as a human cough suppressant and, recently was shown to possess anticancer activity. Regarding to its long its long history of safe usage as an antitussive (cough-suppressing), rapid absorption after oral administration, and apoptosis inducing effect on a variety of cancer cell lines, noscapine is more advantageous in comparison to other natural tubulin-binding anti-cancer compounds, such as the well-established taxanes^4–7^. Additionally, unlike codeine and other opiates, noscapine is neither addictive nor painkilling. This alkaloid accumulates in the cytoplasm or latex of a specialized cells known as laticifers. After morphine, this compound is second abundant alkaloid in the latex^8,9^. According to the literature, stem contains high amount of noscapine in comparison to other tissues. Due to the occurrence of two chiral centers in this molecule, chemical synthesis is prevented and *P. somniferum* is the only commercial source for noscapine^8,10^.

Recently, Winzer and co-workers discovered a co-expressed cluster of 10 genes involved in the noscapine biosynthesis pathway. These genes putatively encode most of the noscapine biosynthetic enzymes^7^. They used a genetic mapping strategy in order to identify a 10-gene cluster representing a 401-kb region of the opium poppy genome in a high-noscapine variety. The BAC sequencing revealed a cluster of 10 physically linked, co-expressed genes for noscapine synthesis. Moreover, co-regulation analysis using *in silico* methods, based on promoter analysis, has been elucidated, and the putative cis/trans regulatory elements represented in the prompter region of these genes have been identified^7,11^. It has been shown that the noscapine pathway starts with the 9-O-methylation of scoulerine catalyzed by scoulerine 9-O-methyltransferase (MT1) and ends with a short-chain dehydrogenase/reductase (NOS or SDR1)^7,9^. In noscapine biosynthesis, in addition, CYP82Y1 has been reported as a first committed step enzyme. This enzyme catalyzes the 1-hydroxylation of N-methylcanadine to 1-hydroxy-N-methylcanadine^12^. Manipulation of each individual gene (MT1, CYP82Y1, and SDR1) has had a significant influence on noscapine accumulation^8,12^. According to a comprehensive study, the accumulation of noscapine has further occurred in the latex. It has also been discovered that SDR1, which catalyzes the final step of noscapine biosynthesis, is localized into latex rather than adjust cells^9,10^. The SDR1 genes have been reported to possess both functions of either dehydrogenase or reductase activity in some species; however, however, in the noscapine biosynthesis pathway, it has only a dehydrogenation function^8,9^.

The exclusive levels and spatiotemporal patterns of expression of the majority of plant genes are assigned to the sophisticated regulation in their cognate promoters. Recent evidences suggest that a variation in the promoter region, particularly close to Translation Start Site (TSS), might affect the gene expression and, subsequently, would have impact on phenotype diversity in human^13^ and plant^14,15^ and secondary metabolite production in plants^16,17^. Promoter divides into two parts; the proximal part where is a central processor of transcription and the distal part where may contain additional regulatory elements such as enhancers and silencers.

Our aim in the present research was to study the gene expression of three determinative genes—MT1, CYP82Y1, and SDR1—involved in the noscapine biosynthesis pathway, and their role within the noscapine content among the collected ecotypes of *P. somniferum* and *P. bracteatum*. Furthermore, the SSR embedded into the promoter region of CYP82Y1, as the first committed step involved in the noscapine biosynthesis pathway, was detected and its impact on the gene expression was assessed. Eventually, the evolution of genes involved in the noscapine pathway—particularly, CYP82Y1 in the Papaveraceae family—was also discussed.

## Results

### CYP82Y1 and MT1 embrace SSR in their promoter region

**Figure 1:**
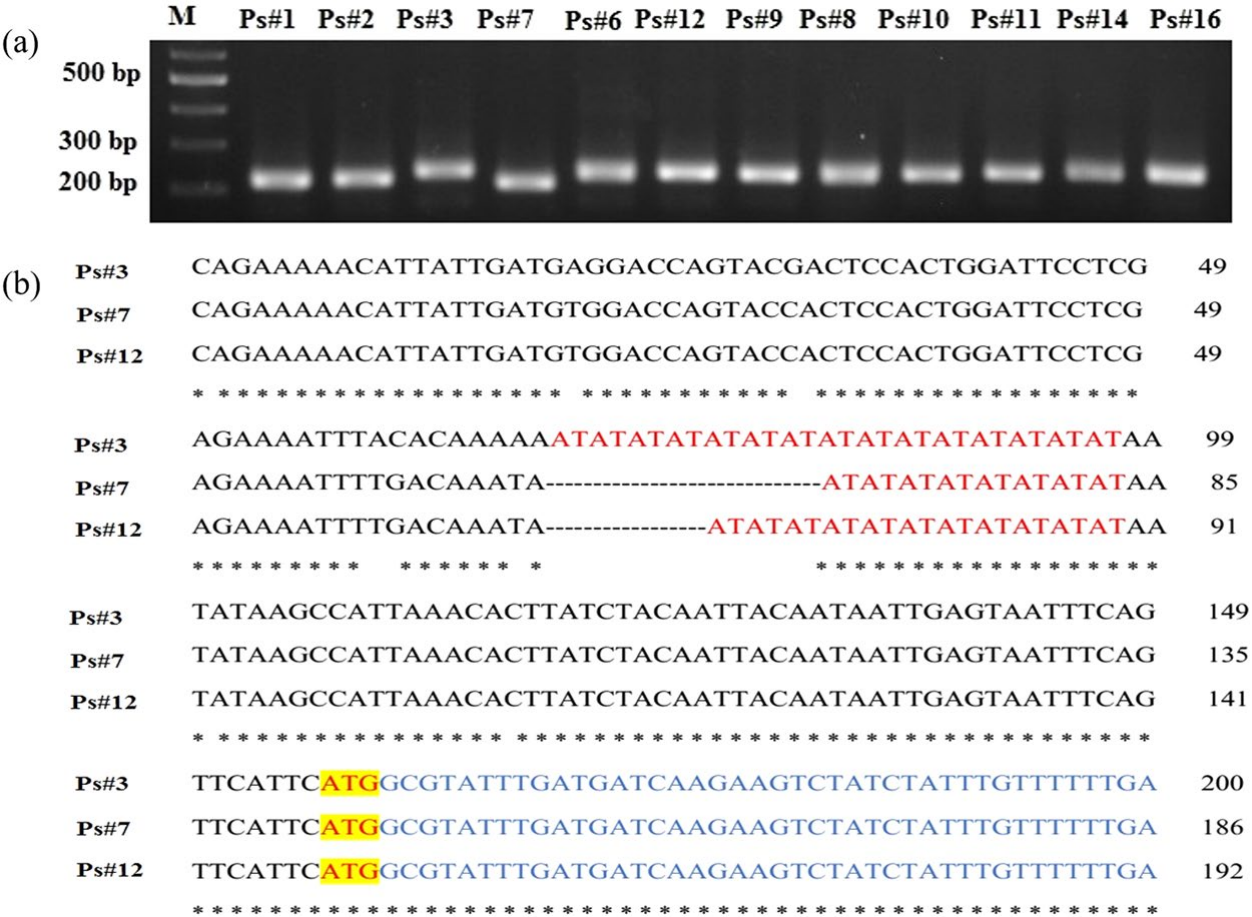
Agarose gel electrophoresis of PCR amplified of detected SSR located in CYP82Y1 upstream region from different collected ecotypes of opium poppy from Iran; (Ps#1-Ps#16) *P. somniferum* L. ecotypes. M; 100 bp DNA ladder (**a**). Sequence alignment of CYP82Y1 promoter region (approximately 200 bp) of the four *P. somniferum* L ecotypes, ‘Ps#7’, ‘Ps#3’, ‘Ps#6’ and ‘Ps#12’. Variation in the number of AT motifs is distinctly observed. AT motifs are colored in red in three ecotypes, which is also considered as a putative TATA-box. The translation start codon (ATG) was also highlighted in yellow. The blue sequences indicates a part of CDS, first exon (**b**).

**Figure 2:**
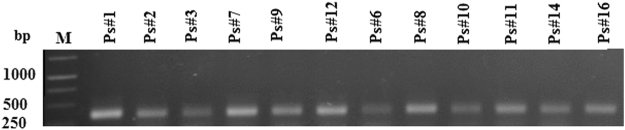
Agarose gel electrophoresis of PCR amplified of detected SSR embedded in upstream region of MT1 gene from different ecotypes of opium poppy; (‘Ps#1’-‘Ps#16’) *P. somniferum* L. ecotypes. M; 250 bp DNA ladder.

By the analysis of 401 Kb obtained from opium poppy^7^, 14 SSRs were earned, scattering in different regions of the 401 Kb of sequenced region (Table 2). Two types of detected SSRs were located in the promoter region of CYP82Y1 [(AT)15] and MT1 [(A)22], taking place in the 59 and the 208 base pairs, just upstream from the ATG codon, respectively. PCR amplification (using genomic DNA as a template) on the specific primer pairs from the 12 *P. somniferum* ecotypes was performed. The difference between amplified products was shown on gel agarose 1.5%. According to the gel electrophoresis, two samples of studied ecotypes named ‘Ps#3’ and ‘Ps#7’ displayed polymorphism in the SSR embedded in promoter region of CYP82Y1 (Fig. 1a,b), whereas no polymorphism was detected in (A)_22_, which was located in the promoter region of MT1 (Fig. 2). The promoter regions of ‘Ps#3’, ‘Ps#7’, ‘Ps#6’ and ‘Ps#12’ were sequenced and the results demonstrated that a significant variation existed in the number of (AT)_n_ microsatellite motifs in the promoter CYP82Y1 of these ecotypes, wherein, ‘Ps#3’ ecotype had [(AT)16], ‘Ps#7’ had [(AT)8], ‘Ps#6’ [(AT)10] and ‘Ps#12’ [(AT)11] motifs (Fig. 1a,b). CYP82Y1 was reported as a first committed step enzyme in the noscapine biosynthesis pathway, therefore, variation in the promoter region of this gene, particularly close to the TSS might influence gene expression.

**Table 1:** Primers used in qPCR, SSR detection and promoter isolation.

Primer no.	Primer name	Use	Primer sequence
1	Spop1_forward_	SSR	5′-TACCACTCCACTGGATTCCTC-3′
2	Spop1_reverse_	SSR	5′-CCTCAAACCCCTCTTCTTCTG-3′
3	Spop2_forward_	SSR	5′-AATGTGTTGGTGAGCACTAGC-3′
4	Spop2_reverse_	SSR	5′-TACACCCCTTGTGGAAAGTGT-3′
5	CYP82Y1-1_forward_	Promoter isolation	5′-CTGGTTGTATCACGGATTTCAC-3′
6	CYP82Y1_reverse_	Promoter isolation	5′-GTGTCAGCAGAAAAGCAAGTATA-3′
7	CYP82Y1-2_forward_	Promoter isolation	5′- AGAACACTTTTGCTTGCTCTTG-3′
8	CYP82Y1-3_forward_	Promoter isolation	5′- CTCCAATCATGTAGGTGAACAAC-3′
9	CYP82Y1-4_forward_	Promoter isolation	5′- GCGTATTTTGTGTTCACTGACA-3′
10	CYP82Y1-5_forward_	Promoter isolation	5′-GTAACGGAAATGAAACAACACTAC-3′
11	PsMT1_forward_	Promoter isolation	5′-GCATCTGGTTGTTCTGTAGTAC-3′
12	PsMT1_reverse_	Promoter isolation	5′-CTGTGGCTGATTGATGATTATGAC-3′
13	SDR1_forward_	Promoter isolation	5′-CCACCTGTTACACATACTATCTTC-3′
14	SDR1_reverse_	Promoter isolation	5′-GAAGGTAGGAAGAAGCTAGTTTG-3′
15	qCYP82Y1_forward_	qRT-PCR	5′-CTATTGCCTTGGACATGTTATC-3′
16	qCYP82Y1_reverse_	qRT-PCR	5′-ATCTACTTCTTCTTTAGCCTTGTC-3′
17	qPsMT1_forward_	qRT-PCR	5′-GTTGGTGGTGGTATTGGTACTT-3′
18	qPsMT1_reverse_	qRT-PCR	5′-ATGCTCTACACCTGGGTATTG-3′
19	qSDR1_forward_	qRT-PCR	5′-GACAGAAAGAGCTTGCCTAAAG-3′
20	qSDR1_reverse_	qRT-PCR	5′-CGACACATCACCTGAAATTATCG-3′
21	Actin_forward_	qRT-PCR	5′-CCAGGCTGTTCAGTCTCTGTAT-3′
22	Actin_reverse_	qRT-PCR	5′-CGCTCGGTAAGGATCTTCATCA-3′

**Table 2:** Detected SSRs embedded into sequenced genome introduced by Winzer *et al*., by running the Perl script MISA (http://pgrc.ipkgatersleben.de/misa/misa.html). The first and second line, which are colored in red, demonstrate the located SSRs in the promoter region of CYP82Y1 and MT1, respectively.

No.	Defined Sequence length	Type	start	end
1	29	(AT)15	237540	237569
2	21	(A)22	355895	355916
3	43	(AT)22	360632	360675
4	19	(T)20	9780	9799
5	23	(T)24	37164	37187
6	33	(TA)17	51682	51715
7	63	(T)64	56838	56901
8	20	(CTTTTAG)3	60229	60249
9	19	(T)20	67811	67830
10	19	(T)20	82607	82626
11	19	(CTTTT)4	131094	131113
12	278	(TTC)93	168005	168283
13	25	(TA)13	213392	213417
14	20	(CAT)7	225030	225050

### Cloning and sequencing of SSR and upstream regions

In order to further understanding of the sequence characteristics of the promoter regions, we isolated the MT1 (using primers 11 and 12) and the SDR1 (using primers 13 and 14) from ‘Ps#7’ ecotype. After conducting PCR, the length of amplified fragments were confirmed using gel agarose (Supplementary Fig. S2A), then, the final products were cloned into pJET vector and subjected to sequencing. Pair-wise alignment between sequenced fragments of MT1 and SDR1 from ‘Ps#7’ (submitted to GenBank as accession number; KY348335 and KY348336, respectively) and reported region from high noscapine variety (HN1), was performed (for alignment details, see Supplementary Fig. S3). Alignment results demonstrate that there were few in/del and point mutations in both promoter regions.

In order to attempt a sequencing of CYP82Y1, we chose samples of ‘Ps#3’ and ‘Ps#7’, which displayed polymorphism within the SSR region in CYP82Y1 promoter, and ‘Ps#12’ as a candidate of the other samples. To this end, we designed some primers (primers 5–10) from different regions of this promoter (Supplementary Fig. S4). After PCR amplification, using the mentioned primers, we amplified the ‘Ps#12’ ecotype (submitted to GenBank as accession number no.: KY348333) using the primers 5 and 6. We also designed primer 5 in close proximity to TSS; this decision was taken because the TSS’s proximity to the ATG seemed to be more conserved than the sequences of the upstream region. The PCR was conducted using primers 5 and 2, and the results showed that amplified fragments differ in size from the presumed promoter region. Regarding the sequencing results, there were several insertions just upstream from the TSS (Supplementary Fig. S7) in the isolated promoter region of CYP82Y1 from ‘Ps#7’ (accession no.: KY348334); this insertion appear neither in the promoter of CYP82Y1 in HN1 nor ‘Ps#12’. The promoter region is frequently divided into two—i.e. the proximal and the distal part. We had assumed that the distal region of CYP82Y1 in this ecotype is highly variable.

### *In silico* analysis of CYP82Y1 promoter region and TATA-box prediction

Computational analysis using PlantCARE database was performed for insertion part of ‘Ps#7’, and several *cis* elements were identified in this part, that could play a role, whether positive or negative, in activity of the CYP82Y1 promoter in this ecotype (Supplementary Table S1). Kinds of TFs have been reported as a regular in BIA production. Silencing and overexpression of CjWRKY, as an example, was determinant in berberine accumulation^18^. Moreover, several putative WRKY and MYB elements placed within or near the promoter regions of reported noscapine gene cluster^7,11^, suggesting that noscapine biosynthesis may be regulated by wRKY and MYB factors. Previously, two TFs (PsWRKY and PsMYB) were isolated from different organ of *P. somniferum* and *P.bracteatum* in our laboratory^11^. Moreover, by utilizing the TRANSFAC and Softberry databases, several TATA-boxes were predicted, indicating that identified SSR is a TATA-box region, with high probability based on its location, which is close to the TSS. A number of studies suggest that the basal level of gene expression is influenced by variations in the TATA-box sequence^19–21^.

### Estimating the effect of (AT)n and (A)n motif number variations on the expression level of the CYP82Y1 and the MT1 genes

**Figure 3:**
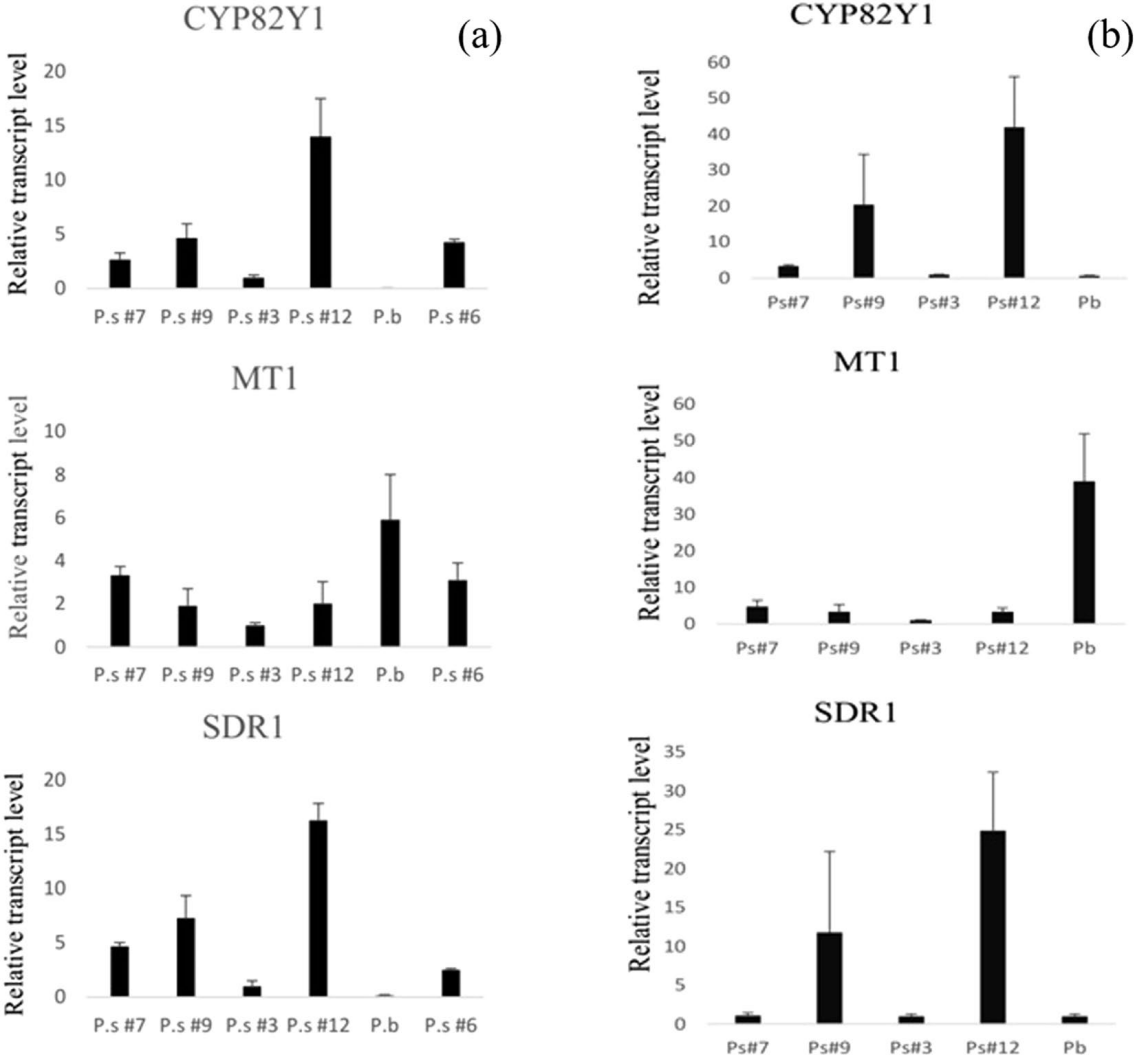
Relative abundance of CYP82Y1, MT1 and SDR1 transcripts in some geographical collected ecotypes of *P.somniferum* L. (‘Ps#3’, ‘Ps#6’, ‘Ps#7’, ‘Ps#9’ and ‘Ps#12’) and *P. bracteatum* from Iran in stem (**a**) and leaf (**b**) tissues.

To understand the impact of detected SSR, which was detected in the upstream region of CYP82Y1 in some ecotypes, on transcripts level, we checked gene expression level by qRT-PCR. The transcript level of stem and leaf from *P. somniferum* ecotypes i.e. -‘Ps#3’, ‘Ps#6’, ‘Ps#7’, ‘Ps#9’ and ‘Ps#12’- and *P. bracteatum* just before anthesis stage were measured. In the selected ecotypes to perform qRT-PCR, ‘Ps#3’, ‘Ps#7’ were picked up as an ecotypes having variation in the ATn motifs number and ‘Ps#6’, ‘Ps#9’ and ‘Ps#12’, which showed no variation. With respect to results shown in Fig. 3, presence of SSR has affected expression of CYP82Y1 gene. According to the quantitative real-time PCR results, remarkably, the expression rate of the CYP82Y1 gene in ecotypes of ‘Ps#6’, ‘Ps#9’ and ‘Ps#12’, which did not show the variation in their promoter region, was more than both ‘Ps#3’ and ‘Ps#7’. For instance, the expression of this gene in Ps#6 is about 2fold compared to Ps# and 4fold compared to Ps#3 ecotype.

In this study, we have also checked the expressions of MT1 and SDR1 as important genes in the noscapine biosynthesis pathway. The transcript level of MT1 in *P. bracteatum* was higher than all of the ecotypes of *P. somniferum* (Fig. 3). This gene converts scoulerine to tetrahydrocolumbamine at high efficiency, and silencing of this gene was associated with accumulation of scoulerine in the latex and capsule^7^. However, the high expression rate of this gene is not related to noscapine content as after this step (conversion of scoulerine to tetrahydrocolumbamine) there are two branch points that are able to change the route of noscapine production to berberine or tetrahydropalmatine. Moreover, the expression of this gene was approximately equal among the *P. somniferum* ecotypes, where there was no variation in their promoter regions of this gene. Another important gene in this pathway is short-chain reductase (SDR), which catalyses the final step of noscapine biosynthesis. The main function of this enzyme takes place in the laticifer and its trace in the noscapine pathway has been recently manifested^9^. It should be noted that the expressions of all the studied genes except MT1 (which has been clarified above) in the *P. bracteatum* was much lesser than the lowest ecotypes of *P. somniferum*.

### Measurement of nocapine content using HPLC

**Figure 4:**
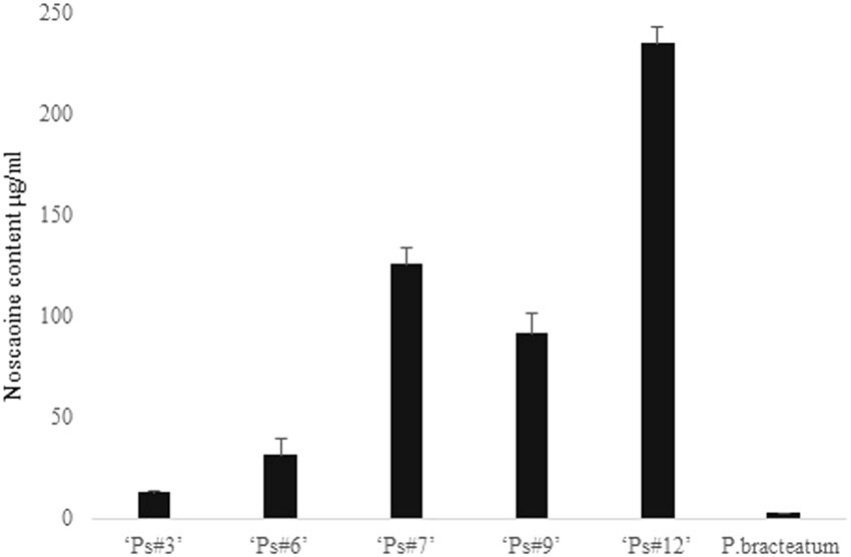
Amount of noscapine in stem tissue from collected ecotypes of *P.somniferum* (‘Ps#3’, ‘Ps#6’, ‘Ps#7’, ‘Ps#9’ and ‘Ps#12’) and *P. bracteatum*.

Noscapine content in the extracted latex from the studied ecotypes was determined using the HPLC method. According to the HPLC results, the amount of noscapine in the studied ecotypes were different. As we had assumed, the content of noscapine in the *P. bracteatum* was less than the *P. somniferum* ecotypes. Noscapine content in the highest ecotype ‘Ps#12’ was 18-fold in comparison to the lowest ecotype ‘Ps#3’ (Fig. 4). It is apparent from the figure that the amount of noscapine in *P. bracteatum* is strikingly lower than all the ecotypes of *P. somniferum*. This species also showed a low rate of expression for both determinative genes, CYP82Y1 and SDR1, in this pathway.

Prior studies have noted the importance of the CYP82Y1 gene in the accumulation of noscapine, and as it is the first committed enzyme in this route, the suppression of CYP82Y1 transcript levels are dramatically reduced in the production of this agent^12^. Analysis of noscapine content revealed that the expression of this gene when it is extreme, could influence noscapine production. In other words, according to Figs 3a and 4, when the transcript level of this gene increase, subsequently, the amount of noscapine also peak and vice versa. For instance, ‘Ps#3’ and ‘Ps#12’ illustrated lowest and highest level in both transcript and metabolite content, respectively. However, there is no such a relation between ecotypes showing intermediate expression of CYP82Y1 gene, suggesting that in order to produce high amount of noscapine, high level expression of this gene is required.

### Evolution of the noscapine biosynthesis pathway in the Papaver genus as the only source of noscapine in the Papaveraceae family

Noscapine is a precious compound with important pharmacological properties; therefore, this agent continues to draw considerable attention. Due to the occurrence of two chiral centers in this molecule, the de novo chemical synthesis is hindered; hence, opium poppy is the only commercial resource of noscapine^8^. Although this agent is substantially produced in *P. somniferum*, biosynthesis in other Papaver genus like *P. bracteatum* occurs. However, the genes that govern this pathway in *P. bracteatum* (which is known as Iranian poppy or Persian poppy) have not been reported. In this study, we analyzed sequence reads obtained from transcriptome projects (accession Nos.; SRX096061, SRX039638). *In silico* assembly of genes involved in this pathway such as; acetyltransferase (AT1), three cytochrome p450s (CYP82Y1, CYP719A21 and CYP82X1), carboxylesterase (CXE1), tetrahydroxyprotoberberine Nmethyltransferase (TNMT), short-chain dehydrogenase/reductase (SDR1) and O-methyltransferases (MT1), suggest that there were a close relationship of noscapine biosynthesis genes in both species (Supplementary Table and Fig. S6(A and B)). We recognized eight genes involved in noscapine biosynthesis pathway in Persian poppy transcriptome data (see Supplementary data S8 to access full length of these identified genes). In addition, for AT1, SDR1, CYP719A21, and CXE1, whose functional motifs and residues had been analyzed^7,12^ were discussed. The results showed that these functional features are present in identified amino acid sequences, which have been obtained from *P. bracteatum*. All of the constructed phylogenic trees and alignments are shown and discussed in Supplementary Fig. S5(A–Z). Prior studies suggested that biosynthesis of this compound is confined to the members of the genus Papaver (Papaveraceae)^8^; nevertheless, using phylogenetic analysis by the exploitation of the transcriptome data set, we found that the enzymes involved in this pathway are likewise limited to this genus. It is to be noted that this analysis takes account of only *P. bracteatum* as a member of the Papaver genus whose Transcriptome data is available.

**Figure 5:**
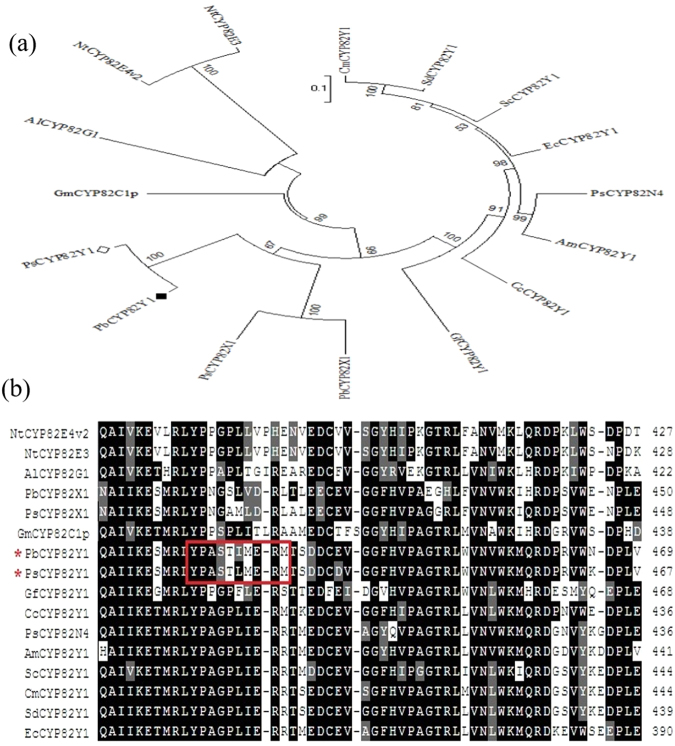
Unrooted neighbor-joining phylogenetic tree for selected CYP82s, constructed using MEGA 7 software. Bootstrap frequencies for each clade were based on 1,000 iterations (**a**). Comparison of substrate recognition site across some CYP82s from Papaveraceae family. The red rectangle represents YPASXXXER conserved motif that is present in PsCYP82Y1 and PbCYP82Y1 (**b**). Abbreviated species names are given before gene identifiers for each protein related to CYP82Y1 are as follows: PbCYP82Y1, *P*. *bracteatum* CYP82Y1; PsCYP82Y1, *P*. *somniferum* (AFB74617); NtCYP82E4v2, *Nicotiana tabacum nicotine* demethylase (ABN42695.1); NtCYP82E3, *N*. *tomentosiformis nicotine* demethylase (ABM46919.1); PsCYP82N4, *P*. *somniferum* (L7X3S1.1); GmCYP82C1p, *Glycine max* cytochrome P450 (AAB94590.1); AlCYP82G1, *Arabidopsis lyrata* DMNT/TMTT homoterpene synthase (XP_002885694.1); PsCYP82X1*, P. somniferum* CYP82X1 (AFB74614); PbCYP82X1, *P. bracteatum* CYP82X1; AmCYP82Y1*, Argemone Mexicana*; CmCYP82Y1, *Chelidonium majus*; CcCYP82Y1, *Corydalis cheilanthifolia*; EcCYP82Y1, *Eschscholzia californica*; GfCYP82Y1, *Glaucium flavum*; ScCYP82Y1, *Sanguinaria canadensis*; SdCYP82Y1, *Stylophorum diphyllum*.

Regarding the investigations, there is a functional motif, YPA(G/S)XXX(E/D)R, which is distinctly present in the CYP82 family^12,22^; however, as far as alignment results are concerned, the motif of YPASXXXER is unique in PsCYP82Y1 and PbCYP82Y1. A notable example is PsCYP82Y1, which displays a remarkable sequence identity with PsCYP82N4 (53%), and accepts N-methylstylopine and N-methylcanadine as substrates. However, while CYP82N4 converted both N-methylcanadine and N-methylstylopine at similar rates, PsCYP82Y1 converted N-methylcanadine with higher efficiency than N-methylstylopine^12,22^. In the noscapine pathway, PsCYP82Y1 converts N-methylcanadine to 1-hydroxy-N-methylcanadine; this step is known as the first committed step in the noscapine biosynthesis pathway. Admittedly, a high rate affinity of PsCYP82Y1 to this substrate would increase the rate of noscapine production. The specific residues for alkaloid binding in PsCYP82N4 are Ile and Leu, whereas the corresponding residues in PsCYP82Y1 are Leu and Ser. The mentioned motif is recognized in Fig. 5 among some CYP82s from some member of the Papaveraceae family. In the frame of this work, we also probed to further identify the genes involved in this pathway from other genus of the Papaveraceae family; however, a low identity in sequence (less than 30 percent sequence identity) was detected. This result demonstrates that these genes are only in the Papaver genus and, therefore, as metabolome analysis proved, the production of noscapine is limited to this genus^8^. In other words, in the other genus of the Papaveraceae family, orthologous noscapine biosynthesis genes were not evolved.

## Discussion

Noscapine is one of the first isolated alkaloids from opium poppy. It has long been used as a cough suppressant, and more recently, it has been investigated as a potential anti-cancer drug. This agent is produced in *P. somniferum* by assisting with some known and unknown genes. This alkaloid accumulate in the cytoplasm or latex and for this metabolite, *P. somniferum* is the only commercial source^6,8^.

This study examines the effect of variation in the promoter region and its impact on gene expression and metabolite content as well as the evolution of the noscapine biosynthesis pathway. Some collected ecotypes of *P. somniferum* and *P. bracteatum* were chosen for this study. The effect of geographical regions on metabolite content and the SSR variation have been reported^14,23^. Several lines of evidence suggest that SSRs are distributed non-randomly across transcribed regions of genomes^24,25^; however, UTRs harbor more SSRs than the coding regions. In particular, 5′UTRs contain a great number of various SSRs that can regulate gene expression^13,16^. In our study, two types of SSRs that were embedded into the CYP82Y1 [(AT)15] and the MT1 [(A)22] promoter regions were detected. CYP82Y1 was known as a fine-tuning enzyme in the noscapine biosynthesis pathway and a variation in its promoter region could influence differentiation in noscapine content as a final product in this pathway^12,16,17^. Two other genes that could influence the accumulation of this compound are MT1, which is responsible for the initiation of this pathway^7^, and SDR1, the catalyzing enzyme of the final step in this pathway^9^. Concrete evidence proved that all of these genes were certainly recruited for the production of noscapine and by silencing each of them the production of this compound would be prevented^7,8,12^. The PCR assay, in order to probe variation in the promoter region, demonstrated variation in two ecotypes (‘Ps#3’ and ‘Ps#7’) for CYP82Y1 (Fig. 1); in contrast, no significant polymorphism was detected for the motif located in the promoter region of MT1 gene. Sequenced promoter region of these genes revealed that there are no significant differences between these regions in ‘Ps#7’ and the HN1 variety. After attempts to isolate the promoter region of CYP82Y1 from ‘Ps#3’ and ‘Ps#7’, we found that this promoter is highly variable when compared with the presumed promoter region of this gene. The isolated promoter region of CYP82Y1 from ‘Ps#7’ demonstrated that there are a fragment insertion in the promoter region of CYP82Y1 in this ecotype. However, a high level of similarity was detected between the promoter region of CYP82Y1 from the ‘Ps#12’ and the HN1 variety. Analysis of experimental results suggested that there are differences in the number of detected SSRs in the collected ecotypes of *P. somniferum*. Moreover, only two ecotypes, ‘Ps#3’ and ‘Ps#7’, exhibit the variation in the SSR region; while others demonstrated no significant differences with each other.

The given results of qRT-PCR revealed that the transcript level of CYP82Y1 in ecotypes, in which their SSR region in the CYP82Y1 promoter region are similar to the HN1 variety, rather than the ecotypes which possess variations in the SSR motif number. We realized that the variation of motif number in the SSR region in two modes, either increases or decreases the motif number, seems to contribute to the decline in the rate of CYP82Y1 gene expression. Studies reported that the presence of variation in the SSR region, specially located in the promoter region, could influence the gene expression, and this impact can be positive or negative. The details of this process differ from organism to organism and from gene to gene^13,14,16,23^. In chickpea, for instance, the presence of SSRs in the 5′UTR region of myo-inositol monophosphatase is associated with expression and metabolite content. Shorter repeats of the SSR in this case had shown two-fold expression in comparison to those that have longer repeats^14^. On the other hand, Kumar and Bhatia have demonstrated that the increase in the motif number of the same SSR in the 5′UTR region of tryptophan decarboxylase in *Catharanthus roseus* L. significantly enhances gene expression^16^. In this work, the expression of the CYP82Y1 gene in ‘Ps#7’, which carried smaller repeats of SSR, was up-regulated to three-folds in comparison to ‘Ps#3’, which had a longer repeat. This experiment was performed in leaf and stem tissues, and we obtained the same results. It should be noted that this SSR is close to TSS (just 59 bp upstream of ATG codon) and its impact on gene expression is logical. However, detected insertion could be a reason for the variation of gene expression. We assumed that because the SSR is near to TSS, this change could influence gene expression rather than that of occurred in distal region. In other words, the (AT)n motif is located in the proximal region, but insertion has occurred in the distal part of this promoter. In this study, we also measured the SDR1 transcript level. The SDR1, which catalyzes the final step of this pathway, forms an integral part in the accumulation of noscapine^8,9^. The expressions of CYP82Y1 and SDR1 in ‘Ps#12’ were more than all the *P. somniferum* collected ecotypes; however, in ‘Ps#3’, these were the lowest. The measuring of noscapine content revealed that the production of this compound in ‘Ps#12’ and ‘Ps#3’ is highest and lowest, respectively, in comparison to other ecotypes. These results confirm that ‘Ps#12’ and ‘Ps#3’ not only are extremes in the transcript level of the CYP82Y1 gene but also are extreme in the noscapine content. Interestingly, the expression level of the CYP82Y1 gene in two ecotypes, ‘Ps#3’ and ‘Ps#7’, was notably less than the rest of ecotypes. On the other hand, intermediate ecotypes in the CYP82Y1 transcript abundance, such as ‘Ps#7’, ‘Ps#6’, and ‘Ps#9’ demonstrated less relation with the noscapine content. A study conducted by Dang and Facchini showed that the Natasha and Marianne chemotypes, which demonstrated high and low amount of noscapine, respectively, had high and low expression of CYP82Y1 gene respectively. However, intermediate chemotypes, including Roxanne and Veronica, had not depicted high relation between CYP82Y1 gene expression and noscapine amount 12. Therefore, it seems that in order to determine an ecotype with high level of noscapine amount, the high expression of the CYP82Y1 gene is the rate limiting step in the production of noscapine compound. In other words, the high level expression of CYP82Y1 gene is vital for higher noscapine production, although, CYP82Y1 enzyme is not the only factor in determining the rate of noscapine production.

Noscapine has a complicated pathway and its production occurs in three cell types. All characterized genes and enzymes involved in the formation of (S)-reticuline, the central branch point intermediate in the biosynthesis of most structural benzylisoquinoline alkaloid subgroups^26^, are localized in companion and sieve cells^8^. It seems that the final conversion of the noscapine catalyst by CXE1 and SDR1 occurs in the latex-bearing laticifers of the adjacent sieve elements^8–10^. In addition, it seems that the acetylation mechanism in this pathway plays a role in the transportation of pathway intermediates^10^. The transportation mechanism of 3-O-acetylpapaveroxine, which is transported from sieve elements to the laticifers, is unknown. Comparing both the transcript and the metabolite levels of studied ecotypes suggested that the accumulation of noscapine in this plant depends on some factors such as the expression of determinative genes, the trafficking of substrates, and the enzymes within the mentioned cells, as well as other presently unknown factors. Further investigations on the transportation mechanism will shed light on trafficking noscapine within opium poppy and the accumulation of this valuable compound^3,10^.

Our study on *P. bracteatum* as a member of the Papaver genus, on which lesser experiments related to noscapine biosynthesis pathway have been performed, demonstrated that this member contains much less noscapine than *P. somniferum*. The expression level of CYP82Y1 and SDR1 confirm metabolite studying results. In the follow-up phase of this study, questions are raised about the production of noscapine in other members of the Papaveraceae family. Our study, which included experimental and *in silico* methods, suggested that although the known genes which participated in noscapine formation were extant in *P. bracteatum*, the expression of these genes, mainly SDR1 and CYP82Y1, was lower than *P. somniferum*. It seems that the expression of these genes is highly restricted by some factors such as their promoter architecture and the presence/absence of specific TFs. In the case of the other members, transcriptome and phylogenetic analyses for all noscapine biosynthesis genes in the seven genera of the Papaveraceae family, whose transcriptome data are accessible on the Sequence Read Archive (SRA) database, suggest that the genes involved in noscapine biosynthesis may not be expressed in these genera or they are absent in the genome of these members. We analyzed the amino acid sequences of some CYP82 families and found that there were some changes in the sequences of these genes, which made PsCYP82Y1 a specific enzyme for this pathway. A conserved motif (YPASXXXER) in the CYP82 families^12,22^ is present only in PsCYP82Y1 and PbCYP82Y1. It is worth mentioning that the Papaver genus is merely the producer of this valuable compound in the Papaveraceae family^8^. We assumed that this motif plays a critical role in this activity, and eventually, in the formation of 1-hydroxy-N-methylcanadine, which is known as a first committed step in the noscapine biosynthesis pathway. It also reported that the variation in this motif influences the affinity rate of this enzyme to the substrate^12^.

Growing the physically-linked clusters of genes for specialized metabolic pathways highlighted an intriguing facet of a plant’s secondary metabolism^27–29^. Although it is a little difficult to reach a consensus on the evolutionary origin of these clusters, it seems that some factors contributed to the formation of gene clusters. Duplication from primary metabolism genes, neofunctionalization, and transposable elements are the likely causes in this process^30–33^. With regard to the transcriptome and metabolome data, noscapine and the 10 genes, which formed the cluster, had been observed in only the Papaver genus and in none of the metabolite and transcripts in the other genera and noscapine-free varieties^7,9^. We hypothesized that the trapping of an ancestral gene that other genes derived from was the reason behind the confinement of the production of this compound in the Papaver genus. This research has opened up many questions in need of future investigations. All these supposed phenomena make the evolutionary Papaver genus the only source of noscapine in the Papaveraceae family and situate *P. somniferum* as a robust source of this valuable compound.

## Material and Method

### Plant material, growth conditions and sample harvesting

In this experiment, 12 seeds of the *P. somniferum* L were used. Ecotypes of opium poppy were collected from different geographical regions of Iran (the region of collected seeds are presented in Supplementary Table S9). These seeds were cultivated in a greenhouse (*Tehran University*) at 20 °C/18 °C (light/ dark) with a photoperiod of (16 h days and 8 h night)^34^. For metabolite and transcript analysis, 5 cm long shoot fragments were harvested immediately under flower bud from each plant stem, 1 to 2 days before anthesis stage^35^.

### Microsatellite mining, DNA extraction and PCR

The Perl script MISA (http://pgrc.ipkgatersleben.de/misa/misa.html) was utilized to detection of SSRs scattered within the partial sequenced poppy genome, which had recently been published. The given sequences had been cloned in Bacterial Artificial Chromosomes by Winzer *et al*.; the promoter region of CYP82Y1 and MT1 genes in BAC179L19 (with accession no.: JQ659009.1) and SDR1 in BAC193L09 (with accession no.: JQ659010.1). The parameters for the SSR search were determined as follows: the size of motif was 1–6 nucleotides and the minimum repeat unit was ten, six and three for mononucleotides, dinucleotides, and all the higher order motifs, respectively, including tri-, tetra-, penta-, and hexanucleotides.

Primers were designed using Primer3 (v. 0.4.0) (http://frodo.wi.mit.edu) and analyzed by Oligo Analyzer (http://eu.idtdna.com/site). Genomic DNA was extracted by the cetyl trimethyl ammonium bromide (CTAB) method and used as the template in the PCR assays. The PCR cycle consisted of an initial denaturation at 95 °C for 5 min followed by 35 cycles of 94 °C for 30 s, 58 °C for 30 s, and 72 °C for 25 s, followed by a final extension of 10 min at 72 °C. Differentiation of amplified samples checked by 1.5% agarose gel electrophoresis.

### Upstream isolation, cloning and sequencing

Ten genes involved in the noscapine biosynthesis pathway had been cloned in four BACs. These BACs, GenBank accession nos. JQ659009 to JQ659012, were used for primer designing and pair-wise alignments as a reference sequence. Subsequently, approximately 1,500 bp upstream of the translation start site, for CYP82Y1, MT1, and SDR1, was isolated as a promoter region. It is worthwhile that the designed primers cover a part of the first exon for the verification of the amplified region. Promoter isolation was performed by the PCR cycle for each promoter region on the collected ecotypes as following program: initial denaturation at 95 °C for 5 min followed by 35 cycles of 94 °C for 30 s, 57 °C for 35 s, and 72 °C for 100 s, and final extension of 10 min at 72 °C. After validation via gel electrophoresis, amplified fragment were purified by using the GenElute™ PCR clean-up Kit (Sigma-Aldrich., Cat No.: NA1020) according to the manufacture’s instruction, and also cloned into pJET1.2/blunt vector (CloneJET PCR Cloning Kit, Fermentas Cat No.: K1231) then subjected to sequencing.

### RNA extraction and quantitative real-time PCR

Stem and leaf (8–16 g) of *P. somniferum* ecotypes (including ‘Ps#3’, ‘Ps#7’, ‘Ps#6’, ‘Ps#9’ and ‘Ps#12’ ecotypes) and *P. bracteatum* were ground to a fine powder under liquid nitrogen. Thereupon, total RNA was extracted using RNeasy Plant Mini Kit (QIAGEN., Cat No.: 74904). Assessment of the extracted RNA integrity was performed by a NanoDrop spectrophotometer (BioTek, EPOCH, serial 121004C, USA), and confirmed by agarose gel electrophoresis. The single strand cDNA synthesis was carried out using Hyperscript^TM^ Reverse Transcriptase (GeneAll Inc, South Korea Cat No.: 601–100) using 1 μg of extracted RNA following the manufacturer’s instruction. To avoid the risk of amplifying possible contaminating genomic DNA, at least one of the primers in each pair was designed to spanning an exon-exon junction. This strategy was used for all three studied genes (i.e. CYP82Y1, MT1 and SDR1) (Supplementary Fig. S2-C1-3). Then, exclusivity performance of designed primers were checked using primer BLAST^36^ and after conducting the PCR, specificity of the primer amplicons were further verified by gel-electrophoretic analysis (Supplementary Fig. S2-C4). The qRT-PCR was performed using BioRad system with the fluorescent dye SYBR®Green Master Mix 2× (Ampliqon, Denmark (Lot No.: A322701)). The qRT-PCR was run at 95 °C for 15 min, 35 cycles at 94 °C 30 s, 58 °C for 20, 72 °C for 15 s. After each run was completed, the dissociation curves were obtained by slowly ramping up the temperature from 65 °C to 95 °C (0.5 °C increase per second) and fluorescence data verified a good specificity of PCR products. Three technical replicates were performed for each sample. For quantifying transcription levels, the reference actin (accession no.: EU531837) was used as an internal control. Cycle thresholds (C(t)s) were analyzed using Livak method (2−ΔΔCt) and relative expression levels were calculated using the Microsoft Excel software (Microsoft Office 2016). Primers are listed in Table 1.

### Noscapine content analysis and chromatographic conditions

Total alkaloid of opium poppy latex were extracted from the latex and approximately 10 µL of exuded latex were re-suspended in 100% methanol for 2 h at room temperature to extract total alkaloid. Extracts were centrifuged for 10 min in 12000 rpm to pellet debris and the supernatants were concentrated by negative pressure. Subsequently, pellets were re-suspended in 50 µL of 100% (v/v) methanol. Noscapine content was determined by using an AZURA high performance liquid chromatography (HPLC) System (KNAUER, Berlin, Germany), diode array detector (DAD 2.1 L), on a Perfectsil® Target ODS-3, 250 mm × 4.6 mm column (5 μm particle size) (MZ-Analysentechnik, Mainz, Germany) with flow rate of 1 mL/min. Chromatographic data were recorded and analyzed by using ClarityChrom® (V. 6.1.0) software. The program was conducted with minor modification based on established method^37^. Separation was achieved using a gradient of solvent A [98% (v/v) H_2_O: 2% (v/v) acetonitrile: 0.04% (v/v) H_3_PO_4_] and solvent B [98% (v/v) acetonitrile: 2% (v/v) H_2_O: 0.04% (v/v) H_3_PO_4_]. Chromatography was initiated in 90% solvent A for 2 min. Subsequently, the gradient was increased to 35% solvent B after 10 min, then increased to 80% solvent B over 5 min.

Noscapine (Temad, Tehran, Iran) quantification were performed using Excel Analysis Tool Pack (Microsoft Office 2016). The calibration curve was constructed based on five different concentrations of noscapine i.e. 5, 12.5, 25, 50 and 60 ug/ml with the following regression equation: y = 150.07 × −28.13. The correlation coefficient (R^2^) was 0.99 for the data sets. Three replications per each sample were injected into the HPLC system.

### Sequence relatedness and evolutionary study of noscapine biosynthesis pathway genes in the Papaveraceae family

Reads sequences from 127 transcriptome sequencing projects of some Papaveraceae members, including *P. bractatum A. mexicana, C. majus*, *C. cheilanthifolia*, *E. californica*, *G. flavum*, *S. canadensis*, *S. diphyllum* were downloaded (http://www.ncbi.nlm.nih.gov/sra/). Afterward, Reads that had high similarity with noscapine biosynthesis pathway genes, i.e. acetyltransferase (AT1), three cytochrome p450s (CYP82Y1, CYP719A21 and CYP82X1), carboxylesterase (CXE1), tetrahydroxyprotoberberine Nmethyltransferase (TNMT), short-chain dehydrogenase/reductase (SDR1) and O-methyltransferases (MT1), were selected using the offline BLASTN 2.6.0 software^38^, and were used to build up consensus sequences. Then, the Read sequences were assembled with the “align-then assemble” strategy employing the Codon Code Aligner v. 5. 0.1. Program (http://www.codoncode.com/aligner/). Then consensus sequences for each group of Reads were created. ORF of each selected sequence was discovered by using the BioEdit software version 7.0.4.1^39^.

Protein Blast was run in order to obtain the proteins that were similar in the amino acid sequence with the query proteins. Meanwhile, multiple-sequence alignment was performed using the web-based Clustal Omega program. The Molecular Evolutionary Genetic Analysis (MEGA) software version 7.0.14 (MEGA, PA, USA) was used in order to implement the maximum likelihood method and, subsequently, the phylogeny tree was constructed using the neighbour-joining (NJ) tree reconstruction method. Bootstrap analysis was performed for each clade based on 1,000 replicates to evaluate the statistical significance of phylogenetic tree nodes. The graphical manipulations of aligned sequences were visualized by using the Sequence Manipulation Suite–Multiple Align Show (http://bioinformatics.org/sms).

## Electronic supplementary material

Supplementary Information
