# Supplementary material for: The effects of promoter variations of the N-Methylcanadine 1-Hydroxylase (CYP82Y1) gene on the noscapine production in opium poppy
Source: Sci Rep. 2018 Mar 21;8:4973. doi: 10.1038/s41598-018-23351-0 (PMC5862900; doi:10.1038/s41598-018-23351-0)
Supplement: Supplementary file 1 — Supplementary Information [file 41598_2018_23351_MOESM1_ESM.pdf]

Title: The effects of promoter variations of the N-Methylcanadine 1-Hydroxylase (CYP82Y1) gene on the noscapine production in opium poppy

Authors: Davar Abedini, Sajad Rashidi Monfared and Alireza Abbasi

"Including all of the tables, figures, gene sequences"

Supplementary Information 1

Table S1. Analysis of putative cis-acting regulatory elements presented in the insertion segment of CYP82Y1 promoter region from Ps#7 ecotype by plantCARE database. In this table cis regulatory elements name and their sequences, positions in the insertion sequences and their function are presented.

**Cis element**	**Position**	**sequence**	**Function**
A-box	+495 and -680	CCGTCC	cis-acting regulatory element
Box I	-407 and -417	TTTCAAA	light responsive element
CAAT-box	+29, +136,+129, +651 and 8 positions	CAAAT	common cis-acting element in promoter and enhancer regions
CCGTCC-box	+495 and 680	CCGTCC	cis-acting regulatory element related to meristem specific activation
CGTCA-motif	+661	CGTCA	cis-acting regulatory element involved in the MeJA-responsiveness
GC-motif	+204	GCCGCGCG	Unknown
GT1-motif	+121	GGTTAA	light responsive element
LAMP-element	+443, +575 and +546	CTTTATCA	part of a light responsive element
MBS	+7	CAACTG	MYB binding site involved in drought-inducibility
Skn-1_motif	+393	GTCAT	cis-acting regulatory element required for endosperm expression
TATA-box	-95, +140, -152, -187 and 15 positions	TATA	core promoter element around -30 of transcription sta[illegible]
TCA-element	+456	CCATCTTTTT	cis-acting element involved in salicylic acid responsiveness
TGACG-motif	-661	TGACG	cis-acting regulatory element involved in the MeJA-responsiveness
Unnamed__13	+134	TCCAAGTATA	Unknown

Supplementary Information 2

Fig. S2-A. Agarose gel electrophoresis of cleand up amplicons of MT1 and SDR1 promoters region from Ps#7 ecotype. Primer used for MT1 were 11 and 12, and for SDR1, 13 and 14 . Primer used also are detailed in Table1. M; 100 bp DNA ladder

Fig. S2-B. Agarose gel electrophoresis of MT1 (B1) and SDR1 (B2) promoter region from different geological collected ecotypes of P. *somniferum* L. No variation was detected into amplified fragments; (Ps#1-Ps#16) *P. somniferum* L. ecotypes. M; 100 bp DNA ladder

Fig. S2-C1. Illustration of designed primers based on exon-exon junction method for Real Time PCR assay. The MT1 gene contains three distinct exons. The forward primer (qMT1-F) was designed on 1st exon and the reverse primer (qMT1-R) cover 1st and 2nd exons.

Fig. S2-C2. Illustration of designed primers based on exon-exon junction strategy for qRT-PCR. The SDR1 gene contains four distinct exons. The forward primer (qSDR1-F) was designed to cover 2nd and 3rd exons and the reverse primer (qSDR1-R) on 3rd exon only.

Fig. S2-C3. Illustration of designed primers based on exon-exon junction strategy for qRT-PCR. The CYP82Y1 gene contains three distinct exons. The forward primer (qCYP82Y1-F) was designed to cover 2nd and 3rd exons and the reverse primer (qCYP82Y1-R) on 3rd exon.

Fig. S2-C4. Primers specificity and amplicons length. The PCR amplification products were analyzed on agarose gel electrophoresis; 1; SDR1, 2; MT1, 3; CYP82Y1, M; 100 bp DNA ladder.

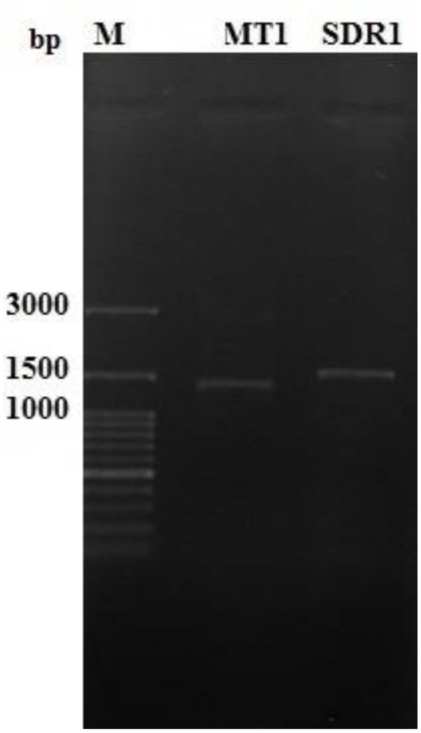

Fig. S2-A.

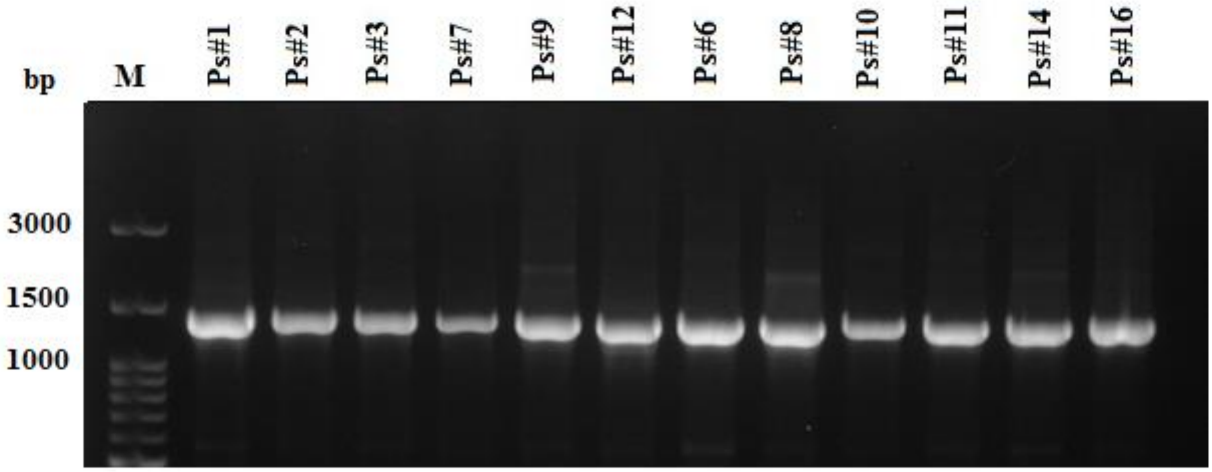
bp
M
Ps#1
Ps#2
Ps#3
Ps#7
Ps#9
Ps#12
Ps#6
Ps#8
Ps#10
Ps#11
Ps#14
Ps#16
3000
1500
1000

Fig. S2-B1.

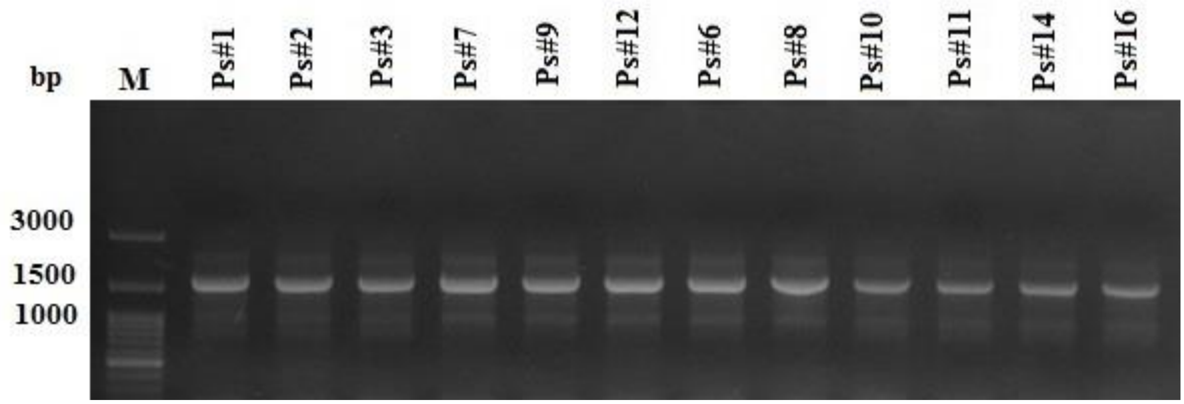

bp
M
Ps#1
Ps#2
Ps#3
Ps#7
Ps#9
Ps#12
Ps#6
Ps#8
Ps#10
Ps#11
Ps#14
Ps#16
3000
1500
1000

Fig. S2-B2.

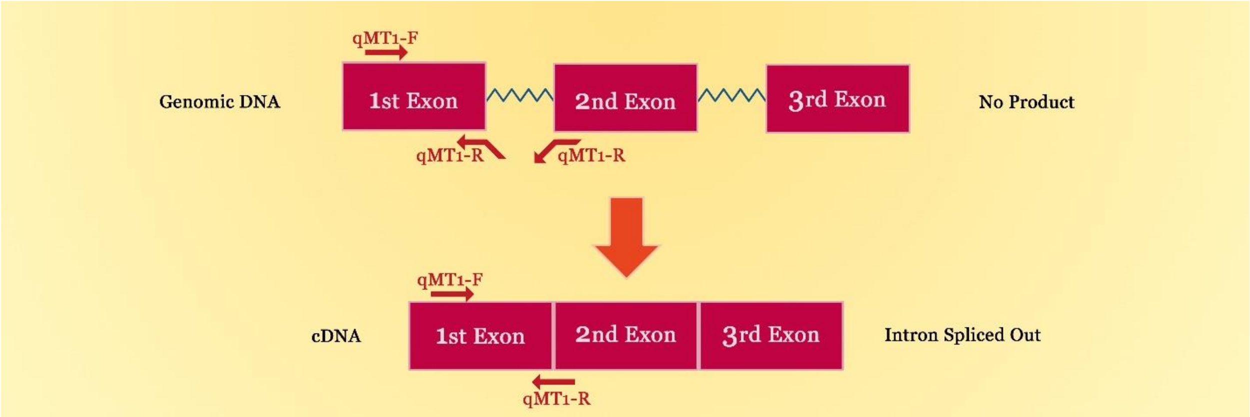
qMT1-F
Genomic DNA
1st Exon
2nd Exon
3rd Exon
No Product
qMT1-R
qMT1-R
qMT1-F
cDNA
1st Exon
2nd Exon
3rd Exon
Intron Spliced Out
qMT1-R

Fig. S2-C1.

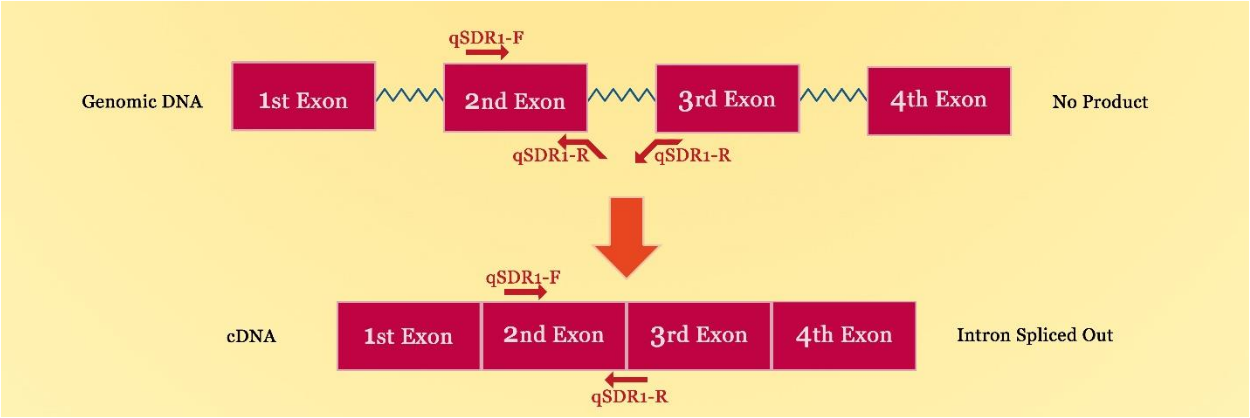
qSDR1-F
Genomic DNA
1st Exon
2nd Exon
3rd Exon
4th Exon
No Product
qSDR1-R
qSDR1-R
qSDR1-F
cDNA
1st Exon
2nd Exon
3rd Exon
4th Exon
Intron Spliced Out
qSDR1-R

Fig. S2-C2.

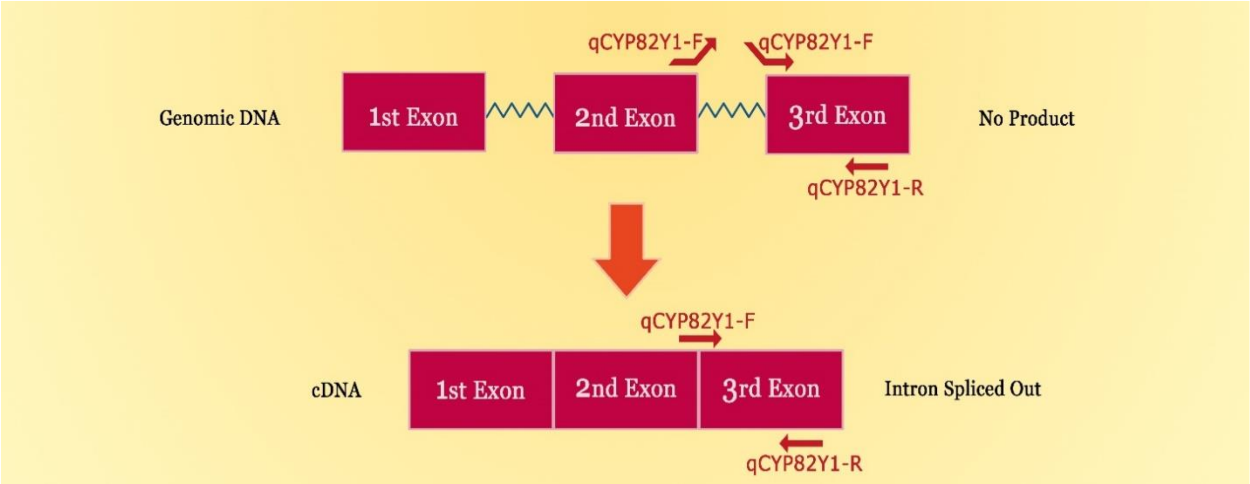

Fig. S2-C3.

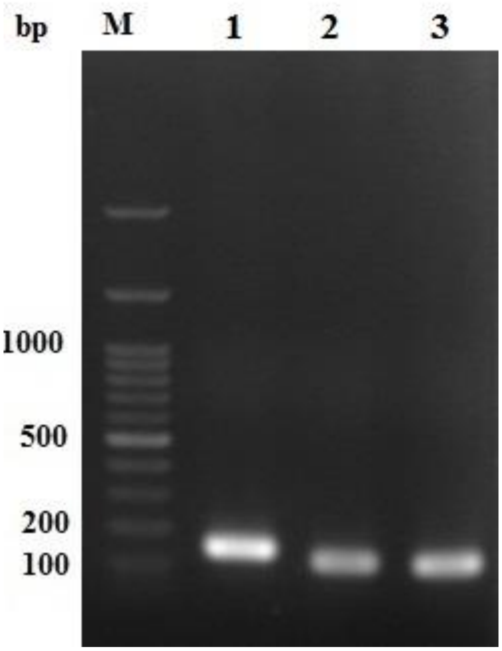

Fig. S2-C4.

Supplementary Information 3

Fig. S3. Pairwise alignment of isolated promoter region of SDR1 (a) and MT1 (b) from Ps#7 and CYP82Y1 (c) from Ps#12, and presumed promoter region of these genes from HN1 variety. High similarity between aligned sequences are visualized utilizing Multiple Alignment Show. The similar nucleotides highlighted in black and the percentage of sequences which must agree for similarity coloring is added 100%.

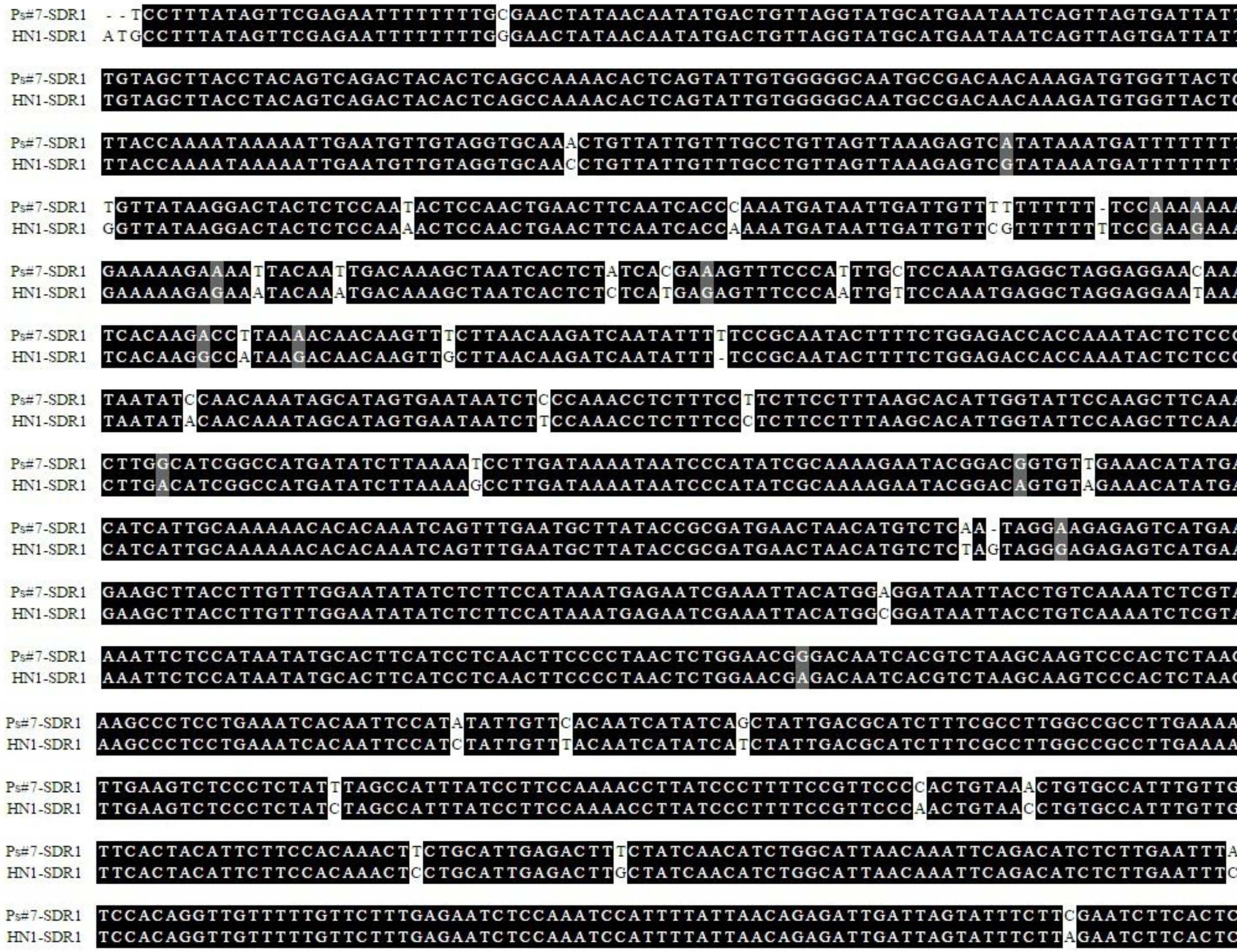

Fig. S3-a

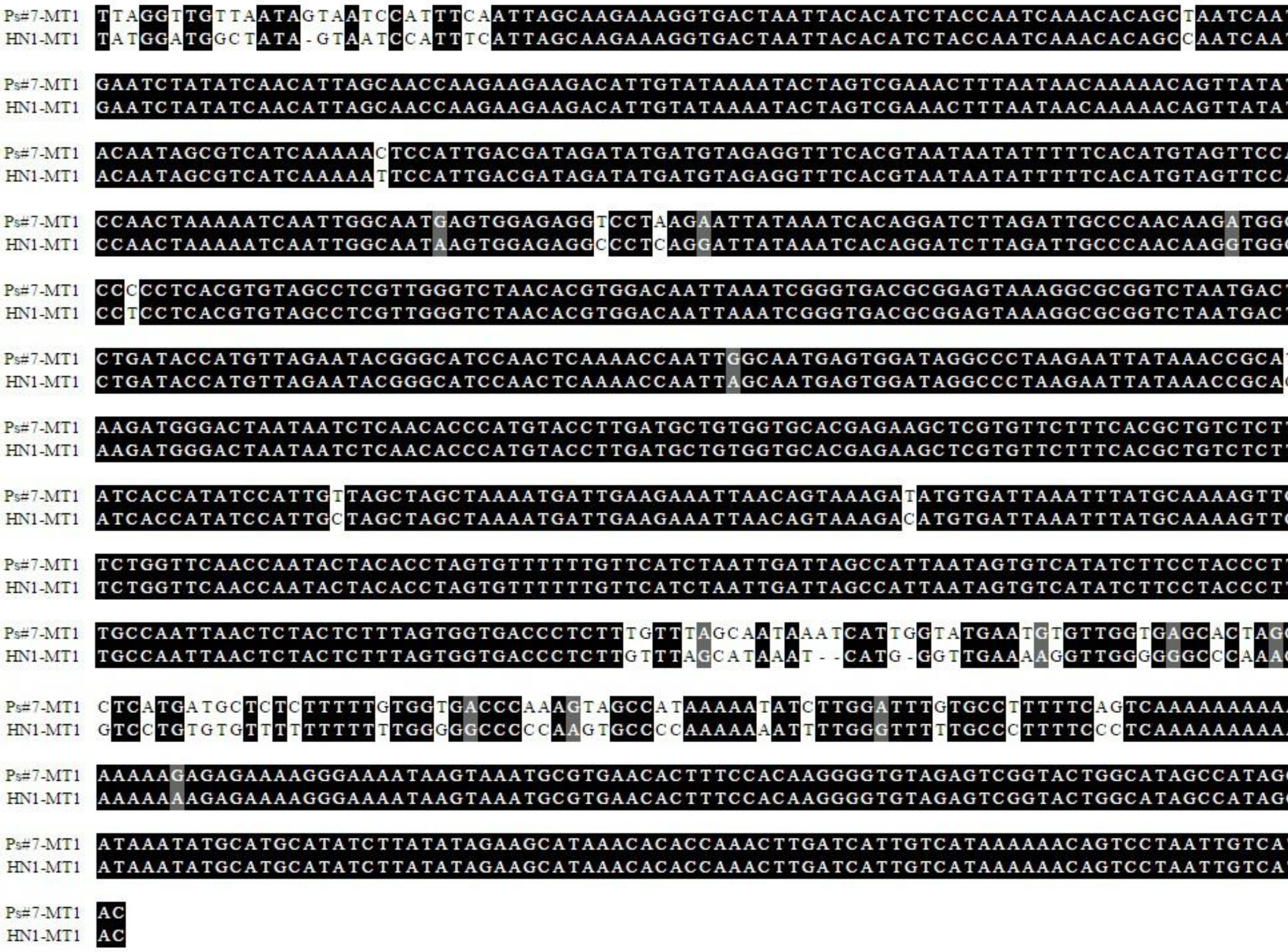

Ps#7-MT1 TTAGGTTGTTAATAGTAATCCATTTCAATTAGCAAGAAAGGTGACTAATTACACATCTACCAATCAAACACAGCTAATCAA
HN1-MT1 TATGGATGGCTATA-GTAATCCATTTCATTAGCAAGAAAGGTGACTAATTACACATCTACCAATCAAACACAGCCAATCAA
Ps#7-MT1 GAATCTATATCAACATTAGCAACCAAGAAGAAGACATTGTATAAAATACTAGTCGAAACTTTAATAACAAAAACAGTTATA
HN1-MT1 GAATCTATATCAACATTAGCAACCAAGAAGAAGACATTGTATAAAATACTAGTCGAAACTTTAATAACAAAAACAGTTATA
Ps#7-MT1 ACAATAGCGTCATCAAAAACTCCATTGACGATAGATATGATGTAGAGGTTTCACGTAATAATATTTTTCACATGTAGTTCC
HN1-MT1 ACAATAGCGTCATCAAAAATTCCATTGACGATAGATATGATGTAGAGGTTTCACGTAATAATATTTTTCACATGTAGTTCC
Ps#7-MT1 CCAACTAAAAATCAATTGGCAATGAGTGGAGAGGTCCTAAGAATTATAAATCACAGGATCTTAGATTGCCCAACAAGATGG
HN1-MT1 CCAACTAAAAATCAATTGGCAATAAGTGGAGAGGCCCTCAGGATTATAAATCACAGGATCTTAGATTGCCCAACAAGGTGG
Ps#7-MT1 CCCCCTCACGTGTAGCCTCGTTGGGTCTAACACGTGGACAATTAAATCGGGTGACGCGGAGTAAAGGCGCGGTCTAATGAC
HN1-MT1 CCTCCTCACGTGTAGCCTCGTTGGGTCTAACACGTGGACAATTAAATCGGGTGACGCGGAGTAAAGGCGCGGTCTAATGAC
Ps#7-MT1 CTGATACCATGTTAGAATACGGGCATCCAACTCAAAACCAATTGGCAATGAGTGGATAGGCCCTAAGAATTATAAACCGCA
HN1-MT1 CTGATACCATGTTAGAATACGGGCATCCAACTCAAAACCAATTAGCAATGAGTGGATAGGCCCTAAGAATTATAAACCGCA
Ps#7-MT1 AAGATGGGACTAATAATCTCAACACCCATGTACCTTGATGCTGTGGTGCACGAGAAGCTCGTGTTCTTTCACGCTGTCTCT
HN1-MT1 AAGATGGGACTAATAATCTCAACACCCATGTACCTTGATGCTGTGGTGCACGAGAAGCTCGTGTTCTTTCACGCTGTCTCT
Ps#7-MT1 ATCACCATATCCATTGTTAGCTAGCTAAAATGATTGAAGAAATTAACAGTAAAGATATGTGATTAAATTTATGCAAAAGTT
HN1-MT1 ATCACCATATCCATTGCTAGCTAGCTAAAATGATTGAAGAAATTAACAGTAAAGACATGTGATTAAATTTATGCAAAAGTT
Ps#7-MT1 TCTGGTTCAACCAATACTACACCTAGTGTTTTTTGTTCATCTAATTGATTAGCCATTAATAGTGTCATATCTTCCTACCCT
HN1-MT1 TCTGGTTCAACCAATACTACACCTAGTGTTTTTTGTTCATCTAATTGATTAGCCATTAATAGTGTCATATCTTCCTACCCT
Ps#7-MT1 TGCCAATTAACTCTACTCTTTAGTGGTGACCCTCTTTGTTTAGCAATAAATCATTGGTATGAATGTGTTGGTGAGCACTAG
HN1-MT1 TGCCAATTAACTCTACTCTTTAGTGGTGACCCTCTTGTTTAGCATAAAT--CATG-GGTTGAAAAGGTTGGGGGCCCAAA
Ps#7-MT1 CTCATGATGCTCTCTTTTTGTGGTGACCCAAAGTAGCCATAAAAATATCTTGGATTTGTGCCTTTTTCAGTCAAAAAAAAA
HN1-MT1 GTCCTGTGTGTTTTTTTTTTTGGGGGCCCCCAAGTGCCCCAAAAAAATTTTGGGTTTTTGCCCTTTTCCCTCAAAAAAAAA
Ps#7-MT1 AAAAAGAGAGAAAAGGGAAAATAAGTAAATGCGTGAACACTTTCCACAAGGGGTGTAGAGTCGGTACTGGCATAGCCATAG
HN1-MT1 AAAAAAAGAGAAAAGGGAAAATAAGTAAATGCGTGAACACTTTCCACAAGGGGTGTAGAGTCGGTACTGGCATAGCCATAG
Ps#7-MT1 ATAAATATGCATGCATATCTTATATAGAAGCATAAACACACCAAACTTGATCATTGTCATAAAAAACAGTCCTAATTGTCA
HN1-MT1 ATAAATATGCATGCATATCTTATATAGAAGCATAAACACACCAAACTTGATCATTGTCATAAAAAACAGTCCTAATTGTCA
Ps#7-MT1 AC
HN1-MT1 AC

Fig. S3-b.

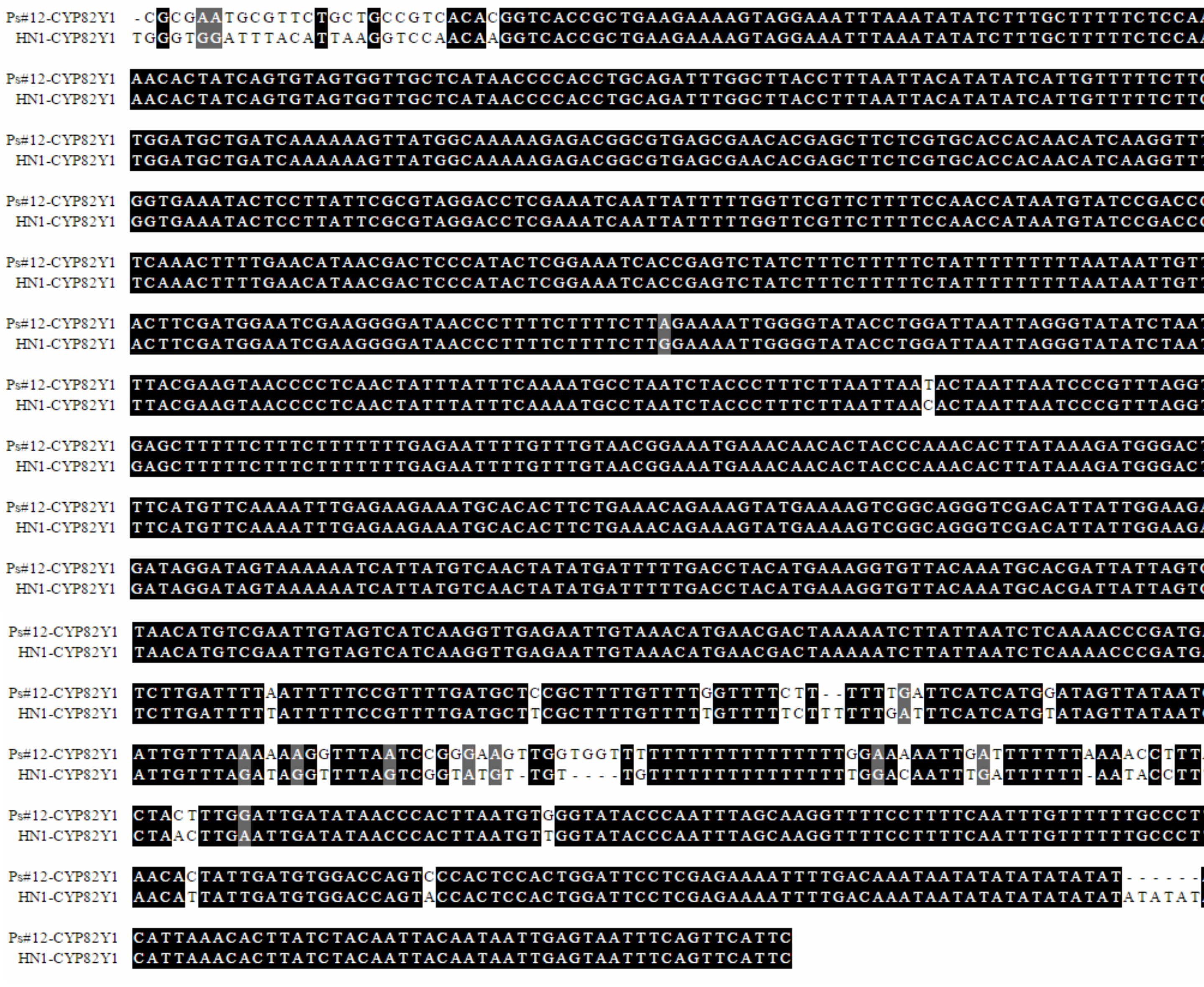
Ps#12-CYP82Y1 -CGCGAATGCGTTCTGCTGCCGTCACACGGTCACCGCTGAAGAAAAGTAGGAAATTTAAATATATCTTTGCTTTTTCTCCA
HN1-CYP82Y1 TGGGTGGATTTACATTAAGGTCCAACAAGGTCACCGCTGAAGAAAAGTAGGAAATTTAAATATATCTTTGCTTTTTCTCCA
Ps#12-CYP82Y1 AACACTATCAGTGTAGTGGTTGCTCATAACCCCACCTGCAGATTTGGCTTACCTTTAATTACATATATCATTGTTTTTCTT
HN1-CYP82Y1 AACACTATCAGTGTAGTGGTTGCTCATAACCCCACCTGCAGATTTGGCTTACCTTTAATTACATATATCATTGTTTTTCTT
Ps#12-CYP82Y1 TGGATGCTGATCAAAAAAGTTATGGCAAAAAGAGACGGCGTGAGCGAACACGAGCTTCTCGTGCACCACAACATCAAGGTT
HN1-CYP82Y1 TGGATGCTGATCAAAAAAGTTATGGCAAAAAGAGACGGCGTGAGCGAACACGAGCTTCTCGTGCACCACAACATCAAGGTT
Ps#12-CYP82Y1 GGTGAAATACTCCTTATTCGCGTAGGACCTCGAAATCAATTATTTTTGGTTCGTTCTTTTCCAACCATAATGTATCCGACC
HN1-CYP82Y1 GGTGAAATACTCCTTATTCGCGTAGGACCTCGAAATCAATTATTTTTGGTTCGTTCTTTTCCAACCATAATGTATCCGACC
Ps#12-CYP82Y1 TCAAACTTTTGAACATAACGACTCCCATACTCGGAAATCACCGAGTCTATCTTTCTTTTTCTATTTTTTTTTAATAATTGT
HN1-CYP82Y1 TCAAACTTTTGAACATAACGACTCCCATACTCGGAAATCACCGAGTCTATCTTTCTTTTTCTATTTTTTTTTAATAATTGT
Ps#12-CYP82Y1 ACTTCGATGGAATCGAAGGGGATAACCCTTTTCTTTTCTTAGAAAATTGGGGTATACCTGGATTAATTAGGGTATATCTAA
HN1-CYP82Y1 ACTTCGATGGAATCGAAGGGGATAACCCTTTTCTTTTCTTGGAAAATTGGGGTATACCTGGATTAATTAGGGTATATCTAA
Ps#12-CYP82Y1 TTACGAAGTAACCCCTCAACTATTTATTTCAAAATGCCTAATCTACCCTTTCTTAATTAATACTAATTAATCCCGTTTAGG
HN1-CYP82Y1 TTACGAAGTAACCCCTCAACTATTTATTTCAAAATGCCTAATCTACCCTTTCTTAATTAACACTAATTAATCCCGTTTAGG
Ps#12-CYP82Y1 GAGCTTTTTCTTTCTTTTTTTGAGAATTTTGTTTGTAACGGAAATGAAACAACACTACCCAAACACTTATAAAGATGGGAC
HN1-CYP82Y1 GAGCTTTTTCTTTCTTTTTTTGAGAATTTTGTTTGTAACGGAAATGAAACAACACTACCCAAACACTTATAAAGATGGGAC
Ps#12-CYP82Y1 TTCATGTTCAAAATTTGAGAAGAAATGCACACTTCTGAAACAGAAAGTATGAAAAGTCGGCAGGGTCGACATTATTGGAAG
HN1-CYP82Y1 TTCATGTTCAAAATTTGAGAAGAAATGCACACTTCTGAAACAGAAAGTATGAAAAGTCGGCAGGGTCGACATTATTGGAAG
Ps#12-CYP82Y1 GATAGGATAGTAAAAAAATCATTATGTCAACTATATGATTTTTGACCTACATGAAAGGTGTTACAAATGCACGATTATTAGT
HN1-CYP82Y1 GATAGGATAGTAAAAAAATCATTATGTCAACTATATGATTTTTGACCTACATGAAAGGTGTTACAAATGCACGATTATTAGT
Ps#12-CYP82Y1 TAACATGTCGAATTGTAGTCATCAAGGTTGAGAATTGTAAACATGAACGACTAAAAATCTTATTAATCTCAAAACCCGATG
HN1-CYP82Y1 TAACATGTCGAATTGTAGTCATCAAGGTTGAGAATTGTAAACATGAACGACTAAAAATCTTATTAATCTCAAAACCCGATG
Ps#12-CYP82Y1 TCTTGATTTTAATTTTTCCGTTTTGATGCTCCGCTTTTGTTTTGGTTTTCTT--TTTTGATTCATCATGGATAGTTATAAT
HN1-CYP82Y1 TCTTGATTTTTATTTTTCCGTTTTGATGCTTCGCTTTTGTTTTTGTTTTTCTTTTTTGATTTCATCATGTATAGTTATAAT
Ps#12-CYP82Y1 ATTGTTTAAAAAAGGTTTAATCCGGGAAGTTGGTGGTTTTTTTTTTTTTTTTTTGGAAAAATTGATTTTTTTAAAACCTTT
HN1-CYP82Y1 ATTGTTTAGATAGGTTTTAGTCGGTATGT-TGT----TGTTTTTTTTTTTTTTTTGGACAATTTGATTTTTT-AATACCTT
Ps#12-CYP82Y1 CTACTTTGGATTGATATAACCCACTTAATGTGGGTATACCCAATTTAGCAAGGTTTTCCTTTTCAATTTGTTTTTTGCCCT
HN1-CYP82Y1 CTAACTTGAATTGATATAACCCACTTAATGTTGGTATACCCAATTTAGCAAGGTTTTCCTTTTCAATTTGTTTTTTGCCCT
Ps#12-CYP82Y1 AACACTATTGATGTGGACCAGTCCCACTCCACTGGATTCCTCGAGAAAATTTTGACAAATAATATATATATATAT------
HN1-CYP82Y1 AACATTATTGATGTGGACCAGTACCACTCCACTGGATTCCTCGAGAAAATTTTGACAAATAATATATATATATATATATAT
Ps#12-CYP82Y1 CATTAAACACTTATCTACAATTACAATAATTGAGTAATTTCAGTTCATTC
HN1-CYP82Y1 CATTAAACACTTATCTACAATTACAATAATTGAGTAATTTCAGTTCATTC

Fig. S3-c.

Supplementary Information 4

Fig. S4. The location of designed primers to isolation of CYP82Y1 upstream region. Primer sequences are colored in red and the arrows indicate the primer direction, the SSR region are highlighted in green and the start codon (ATG) are highlighted in yellow. Primers are detailed in the Table 1.

CGTCCTGCTCTTAGGGAGGAAAATCAAAGCTAGCCCTGTAAGGAATACTAGTATATG

PICYP82Y1-2

TGGTCGGTGCTGATATCTTGTGAGAACACTTTTGCTTGCTCTTGCTTGGAATTGGATT

TGTAGTTACCCTGAATTAAAAAAGTAGAGCAACAAAAATGTGTAATTTCGTTATTTT

PICYP82Y1-4

TCGCGTATTTTGTGTTCACTGACATTATTTCCAATATGTATGCAAGTTTAGAGCTTGA

TTTGCTTAGATTCAGATTGCACTTACTGGTGCTAGTAAAATTGTAGGTAAACAAACA

CTGTTTTGATTAGGTAAATTTGTGGTGAAAACTCGTAGAAAAGATCACAGTGTTCTC

TAGGGTGTTCCAAAGCCAACACCTATAGATCATACATTTTGATCACACGTTAATTAA

PICYP82Y1-1

CAAAATCTCTGATCCGCATCTCCAAATAAAATTTACTGGTTGTATCACGGATTTCAC

CCATGTGGGTGGATTTACATTAAGGTCCAACAAGGTCACCGCTGAAGAAAAGTAGG

PICYP82Y1-3

AAATTTAAATATATCTTTGCTTTTTCTCCAATCATGTAGGTGAACAACAAACACTATC

AGTGTAGTGGTTGCTCATAACCCCACCTGCAGATTTGGCTTACCTTTAATTACATATA

TCATTGTTTTTCTTCAATCATTTTAGCTACTAATGGATGCTGATCAAAAAAGTTATGG

CAAAAAGAGACGGCGTGAGCGAACACGAGCTTCTCGTGCACCACAACATCAAGGTT

TATGGAGATTAACTACTTGGGTGAAATACTCCTTATTCGCGTAGGACCTCGAAATCA

ATTATTTTTGGTTCGTTCTTTTCCAACCATAATGTATCCGACCCATATTCGTTTGTTGC

ACCTCAAACTTTTGAACATAACGACTCCCATACTCGGAAATCACCGAGTCTATCTTT

CTTTTTCTATTTTTTTTTAATAATTGTTCTTGAAGTGTGGATGGTCACTTCGATGGAAT

CGAAGGGGATAACCCTTTTCTTTTCTTGGAAAATTGGGGTATACCTGGATTAATTAG

GGTATATCTAATAAGAAAAAAATCCGGATCATTACGAAGTAACCCCTCAACTATTTAT

TTCAAAATGCCTAATCTACCCTTTCTTAATTAACACTAATTAATCCCGTTTAGGTTAA

TTAATTTAAATTTTTGAGCTTTTTCTTTCTTTTTTTGAGAATTTTGTTTGTAACGGAAA

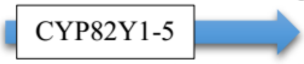

TGAAACAACACTACCCAAACACTTATAAAGATGGGACTGTAAAGCTATTGAGAGAT

TTCATGTTCAAAATTTGAGAAGAAATGCACACTTCTGAAACAGAAAGTATGAAAAG

TCGGCAGGGTCGACATTATTGGAAGATAATGACCACAAATAGCCGATAGGATAGTA

AAAAATCATTATGTCAACTATATGATTTTTGACCTACATGAAAGGTGTTACAAATGC

ACGATTATTAGTCGGCAATATATTAATTTCATAACATGTCGAATTGTAGTCATCAAG

GTTGAGAATTGTAAACATGAACGACTAAAAATCTTATTAATCTCAAAACCCGATGAT

CCAAATTTTCAATTTTTTCTTGATTTTTATTTTTCCGTTTTGATGCTTCGCTTTTGTTTT

TGTTTTTCTTTTTTGATTTCATCATGTATAGTTATAATCTTGAAGAAAGATTTGAAAA

TTGTTTAGATAGGTTTTAGTCGGTATGTTGTTGTTTTTTTTTTTTTTTTGGACAATTTG

ATTTTTTAATACCTTTAAAACACCCCTTAGCCCACTAACTTGAATTGATATAACCCAC

TTAATGTTGGTATACCCAATTTAGCAAGGTTTTCCTTTTCAATTTGTTTTTTGCCCTTT

Spop1-F

TCAAAAAAGTTCAGAAAAACATTATTGATGTGGACCAGTACCACTCCACTGGATTCC

TCGAGAAAATTTTGACAAATAATATATATATATATATATATATATATATATATATAATATA

TSS

AGCCATTAAACACTTATCTACAATTACAATAATTGAGTAATTTCAGTTCATTCATGG

CGTATTTGATGATCAAGAAGTCTATCTATTTGTTTTTTGATCAACCAACTGCAGTTGG

CYP82Y1-R

CACTCTTATACTTGCTTTTCTGCTGACACTTTCGCCTGTTATTATTTACTATGAACAG

Spop1-R

AAGAAGAGGGGTTTGAGGCGAAATCGCACCGCAATTACAACGACTCCAT

Supplementary Information 4

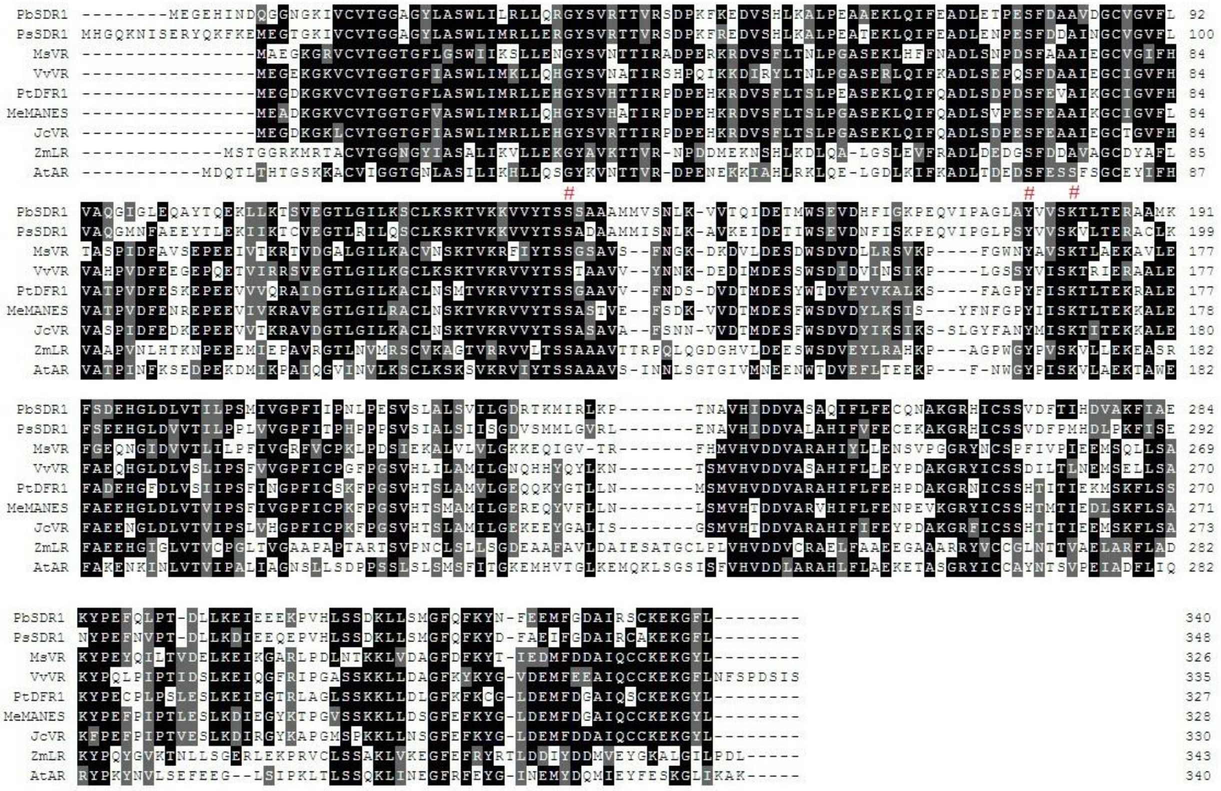

Fig.S5-A1. Sequence alignment of the amino acid sequence of identified SDR1 from *P. bracteatum* . Residues highlighted in black are identical and those in dark gray are similar. Percentage of sequences which must agree for identity or similarity coloring to be added 50%. Hyphens represent gaps inserted for optimal alignment. Catalytic residues (Serine (S), Tyrosine (Y) and lysine (K)) are indicated above the alignment by red # (hash key). These residues are conserved in all the protein sequences and their catalytic functions have recently reported.

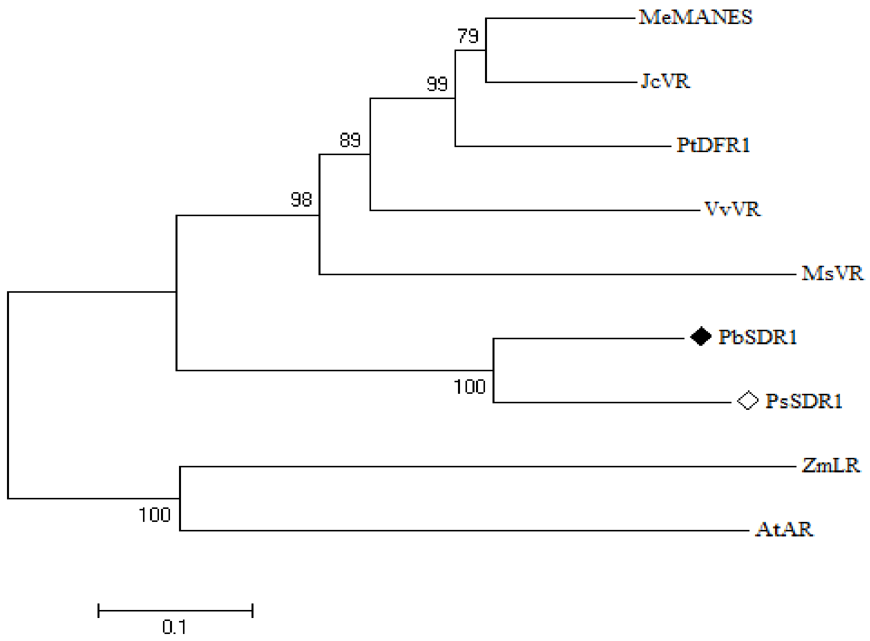

Fig.S5-A2. Unrooted neighbor-joining phylogenetic tree for selected SDR1 consensus sequence exploiting RNA-seq data obtained from *Papaver bracteatum* genus from a BLASTP search of the NCBI non-redundant protein database, constructed using MEGA 7 software. Bootstrap frequencies for each clade were based on 1,000 iterations.

Abbreviated species names are given before gene identifiers for each protein related to SDR1 are as follows:

PbSDR1, *P. bracteatum* short-chain dehydrogenase/reductase; PsSDR1, *P. somniferum* short-chain dehydrogenase/reductase (AFB74619.1); PbSalR, *P. bracteatum* salutaridine reductase (ABO93462.1); MeMANES, *Manihot esculenta* MANES_01G266200 (OAY62412); VvVR, *Vitis vinifera* vestitone reductase isoform X3 (XP_002278819); PtDFR1, *Populus trichocarpa* 2'-hydroxy isoflavone/dihydroflavonol reductase (XP_002301290); JcVR, *Jatropha curcas* vestitone reductase (XP_01208357); AlDFR, *Arabidopsis lyrata* dihydroflavonol 4-reductase (XP_002882011); AtND, *A. thaliana* NAD(P)-binding Rossmann-fold superfamily protein (NP_191681); ZmLR, *Zea mays* leucoanthocyanidin reductase (NP_001148881); MsVR, *Medicago sativa* vestitone reductase (AAB41550); ZmSDR, *Z. mays* short-chain dehydrogenase/reductase (NP_001148440); AtAR, *A. thaliana* anthocyanidin reductase (NP_176365)

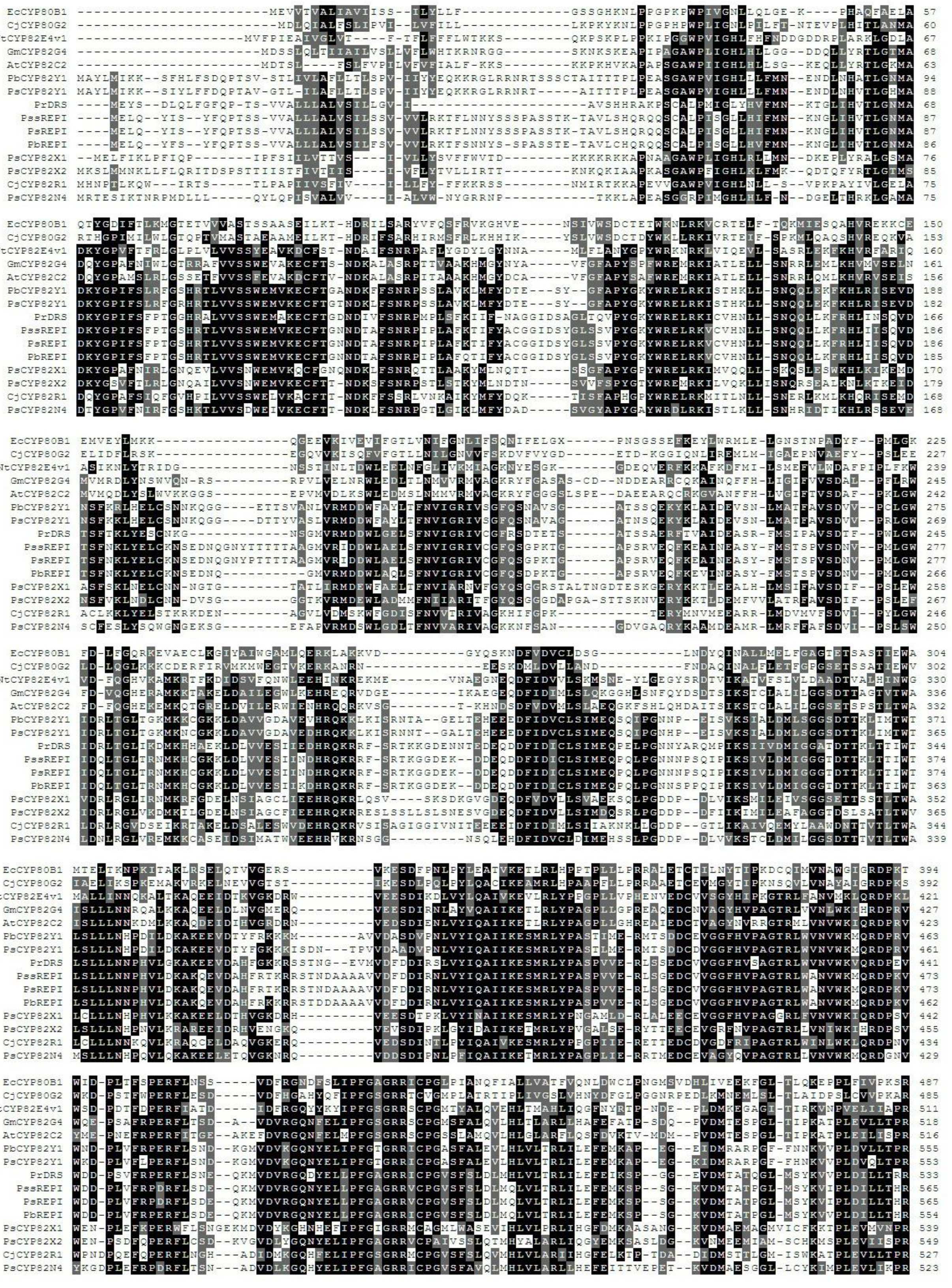

Fig.S5-B1. Sequence alignment of the amino acid sequence of identified CYP82Y1 from *P. bracteatum* . Residues highlighted in black are identical and those in dark gray are similar. Percentage of sequences which must agree for identity or similarity coloring to be added 50%. Hyphens represent gaps inserted for optimal alignment.

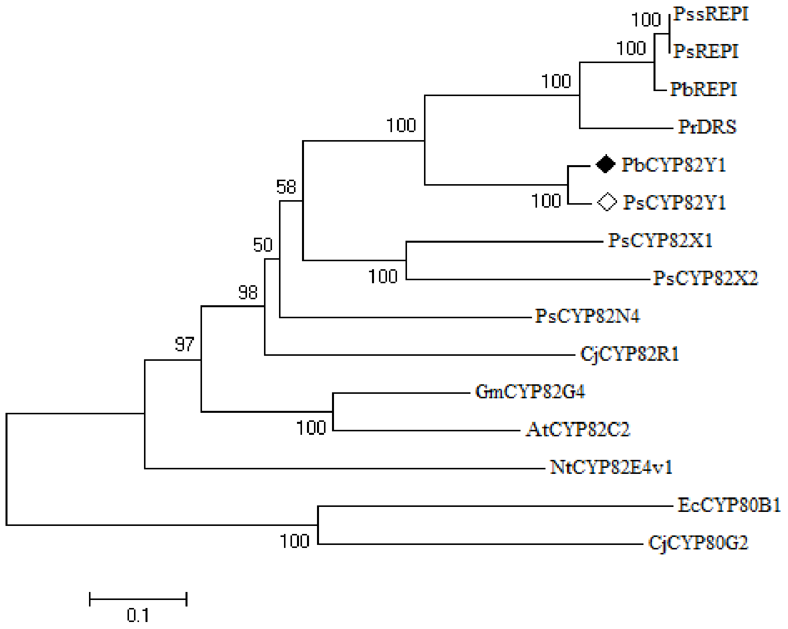

Fig.S5-B2. Unrooted neighbor-joining phylogenetic tree for CYP82Y1 selected consensus sequence exploiting RNA-seq data obtained from *Papaver bracteatum* genus from a BLASTP search of the NCBI non-redundant protein database, constructed using MEGA 7 software. Bootstrap frequencies for each clade were based on 1,000 iterations. Abbreviated species names are given before gene identifiers for each protein related to CYP82Y1 are as follows:

PbCYP82Y1, *P. bracteatum* CYP82Y1; PsCYP82Y1, *P. somniferum* (AFB74617); PssREPI, *P. somniferum* (subsp. Setigerum) reticuline epimerase (AKO60180); PsREPI, *P. somniferum* reticuline epimerase (AKO60175); PbREPI, *P. bracteatum* reticuline epimerase (AKO60179); PrDRS, *P. rhoeas* 1,2-dehydroreticuline synthase (AKO60176); PsCYP82X1, *P. somniferum* CYP82X1 (AFB74614); PsCYP82X2, *P. somniferum* CYP82X2 (AFB74616); CjCYP82R1, *C. japonica* var. dissecta CYP82R1 (BAF98472); PsCYP82N4, *P. somniferum* (S)-cis-N-methylstylopine 14-hydroxylase (AGC92398.1); EcCYP80B1, *E. californica* (S)-N-methylcoclaurine 3'-hydroxylase (AAC39453.1); CjCYP80G2, *C. japonica* var. dissecta] corytuberine synthase (BAF80448.1); NtCYP82E4v1, *N. tabacum* CYP82E4v1 (DQ131886); GmCYP82G4, *G. max* CYP82G4 (XP_003523888); AtCYP82C2, *A. thaliana* CYP82C2 (O49394)

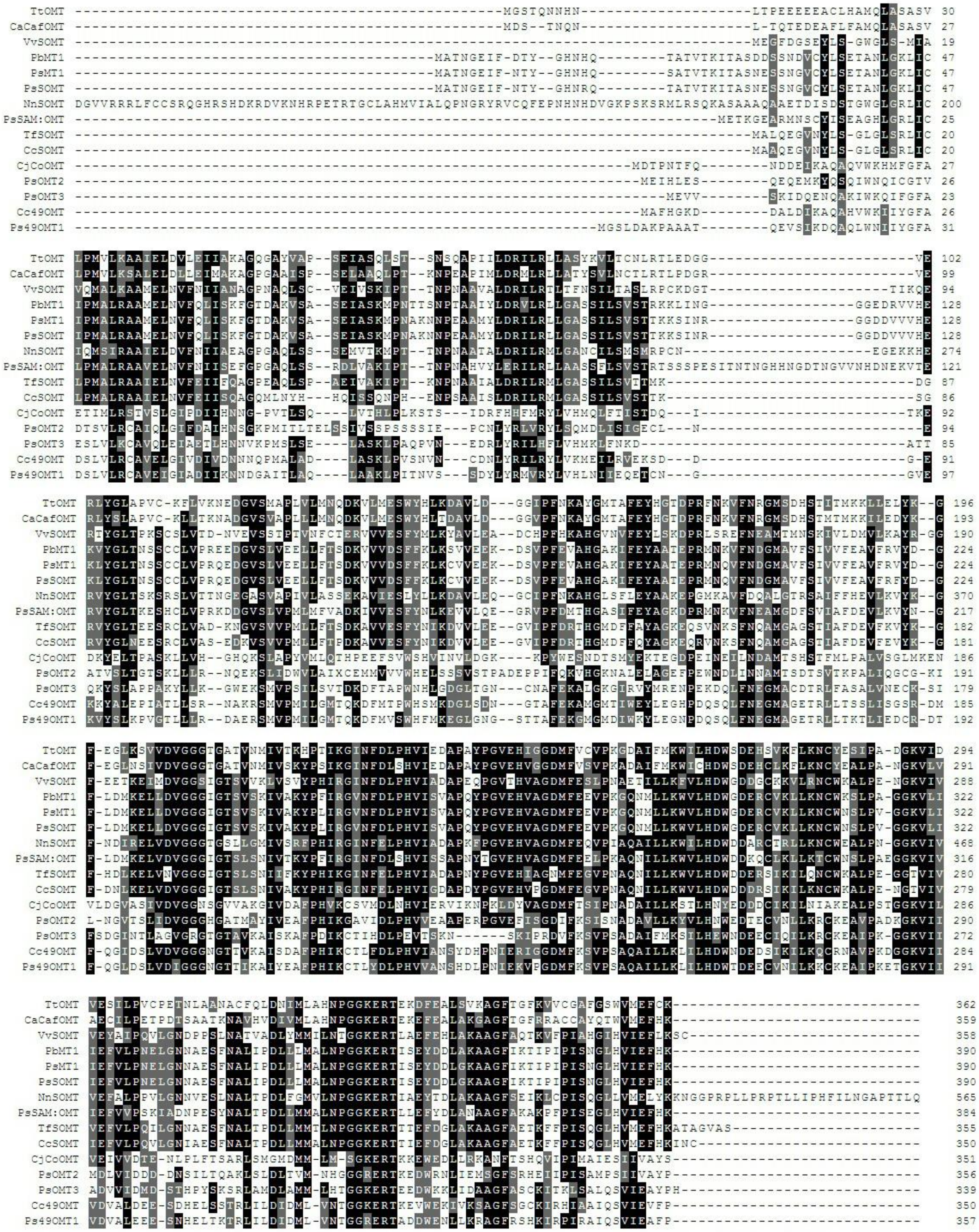

Fig.S5-C1. Sequence alignment of the amino acid sequence of identified MT1 from *P. bracteatum* . Residues highlighted in black are identical and those in dark gray are similar. Percentage of

sequences which must agree for identity or similarity coloring to be added 50%. Hyphens represent gaps inserted for optimal alignment.

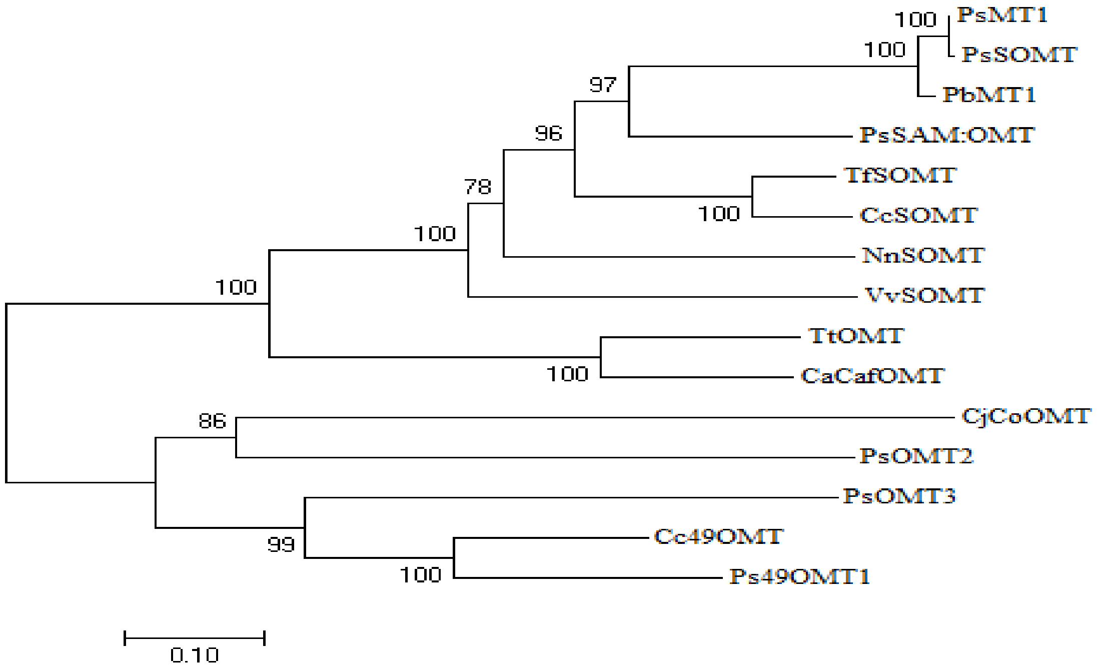

Fig.S5-C2. Unrooted neighbor-joining phylogenetic tree for MT1 selected consensus sequence exploiting RNA-seq data obtained from *Papaver bracteatum* genus from a BLASTP search of the NCBI non-redundant protein database, constructed using MEGA 7 software. Bootstrap frequencies for each clade were based on 1,000 iterations. Abbreviated species names are given before gene identifiers for each protein related to MT1 are as follows:

PbMT1, *P. bracteatum* O-methyltransferase 1; PsMT1, *P. somniferum* O-methyltransferase 1 (AFB74611); PsSOMT, *P. somniferum* scoulerine-9-O-methyltransferase (AFK73709); PsSAM:OMT, *P. somniferum* S-adenosyl-L-methionine:O-methyltransferase (AKO60157); TfSOMT, *Thalictrum flavum* (S)-scoulerine 9-O-methyltransferase (AAU20770); CcSOMT, *Coptis chinensis* (S)-scoulerine 9-O-methyltransferase (ACL31653); NnSOMT, *Nelumbo nucifera* (S)-scoulerine 9-O-methyltransferase-like (XP_010242195); VvSOMT, *Vitis vinifera* (S)-scoulerine 9-O-methyltransferase (XP_002280092); CjCoOMT, *Coptis japonica* Columbamine O-methyltransferase (Q8H9A8.1); TtOMT, *Thalictrum tuberosum* O-methyltransferase (AAD29845.1); PsOMT2, *P. somniferum* narcotoline-O-methyltransferase (AFK73710.1); PsOMT3, *P. somniferum* O-methyltransferase (AFK73711.1); Cc49OMT, *Coptis chinensis* 3′-hydroxy-N-methylcoclaurine 4′OMT (ABY75613.1); Ps49OMT1, *P. somniferum* 3′-hydroxy-N-methylcoclaurine 4′OMT2 (AAP453141); CaCafOMT, Capsicum annuum caffeate O-methyltransferase (AAG43822.1)

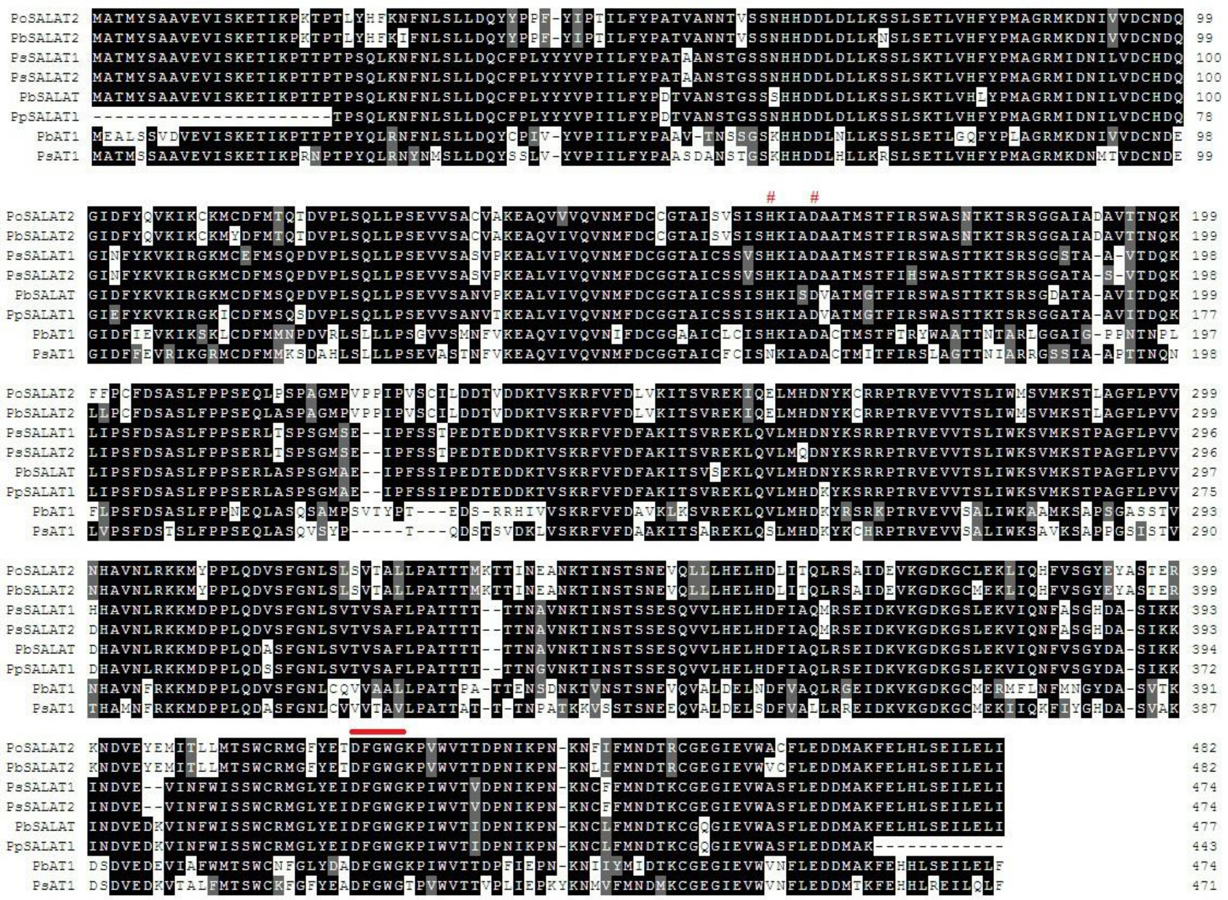

Fig.S5-D1. Sequence alignment of the amino acid sequence of identified AT1 from *P. bracteatum* . Residues highlighted in black are identical and those in dark gray are similar. Percentage of sequences which must agree for identity or similarity coloring to be added 50%. Hyphens represent gaps inserted for optimal alignment. The position of the highly conserved histidine and aspartate of the HxxxD motif are indicated above the alignment by # (hash key). The highly conserved histidine is substituted by asparagine in the PsAT1 sequence. However, this residue is conserved in PbAT1. The red line above the alignment indicates the position of the highly conserved DFGWG motif which together with the HxxxD motif is a feature of BAHD-type acyltransferases.

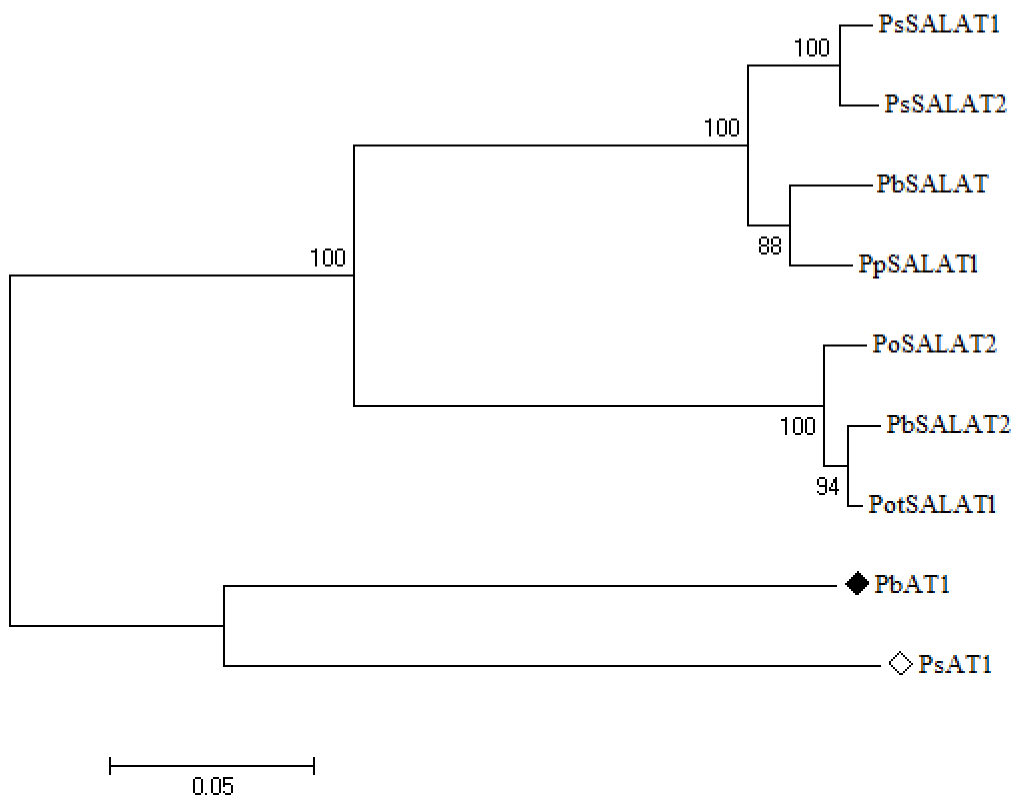

Fig.S5-D2. Unrooted neighbor-joining phylogenetic tree for AT1 selected consensus sequence exploiting RNA-seq data obtained from *Papaver bracteatum* genus from a BLASTP search of the NCBI non-redundant protein database, constructed using MEGA 7 software. Bootstrap frequencies for each clade were based on 1,000 iterations.

Abbreviated species names are given before gene identifiers for each protein related to AT1 are as follows:

PbAT1, *P. bracteatum* acetyltransferase 1; PsAT1, *P. somniferum* acetyltransferase 1 (AFB74620); PbSALAT, P. *bracteatum* salutaridinol 7-O-acetyltransferase (ACI45392.1); PsSALAT1, *P. somniferum* salutaridinol 7-O-acetyltransferase (ACI45390.1); PsSALAT2, *P. somniferum* salutaridinol 7-O-acetyltransferase (BAT70024.1); Posal, *P. orientale* salutaridinol 7-O-acetyltransferase (ACI45395.1); Pbsal2, *P. bracteatum* salutaridinol 7-O-acetyltransferase (ACI45393.1); P-posal, *P. pseudo-orientale* salutaridinol 7-O-acetyltransferase, partial (AKN62840.1); EgVS, *Eucalyptus grandis* vinorine synthase-like (XP_010044504)

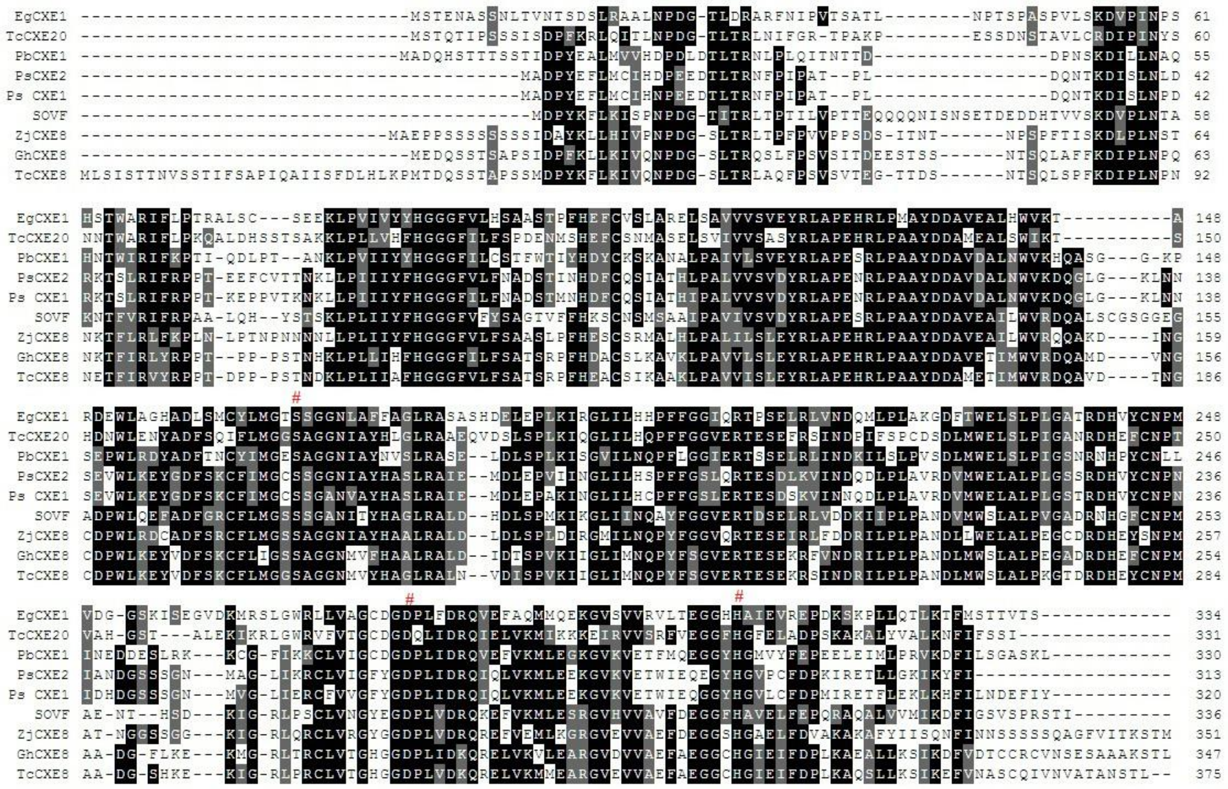

Fig.S5-E1. Sequence alignment of the amino acid sequence of identified CXE1 from *P. bracteatum* and its homologues in *P. somniferum*. Residues highlighted in black are identical and those in dark gray are similar. Percentage of sequences which must agree for identity or similarity coloring to be added 50%. Hyphens represent gaps inserted for optimal alignment. Catalytic residues (Serine (S), Aspartic acid (D) and Histidine (H)) are indicated above the alignment by red # (hash key). These residues are conserved in all the protein sequences and their catalytic functions have recently reported.

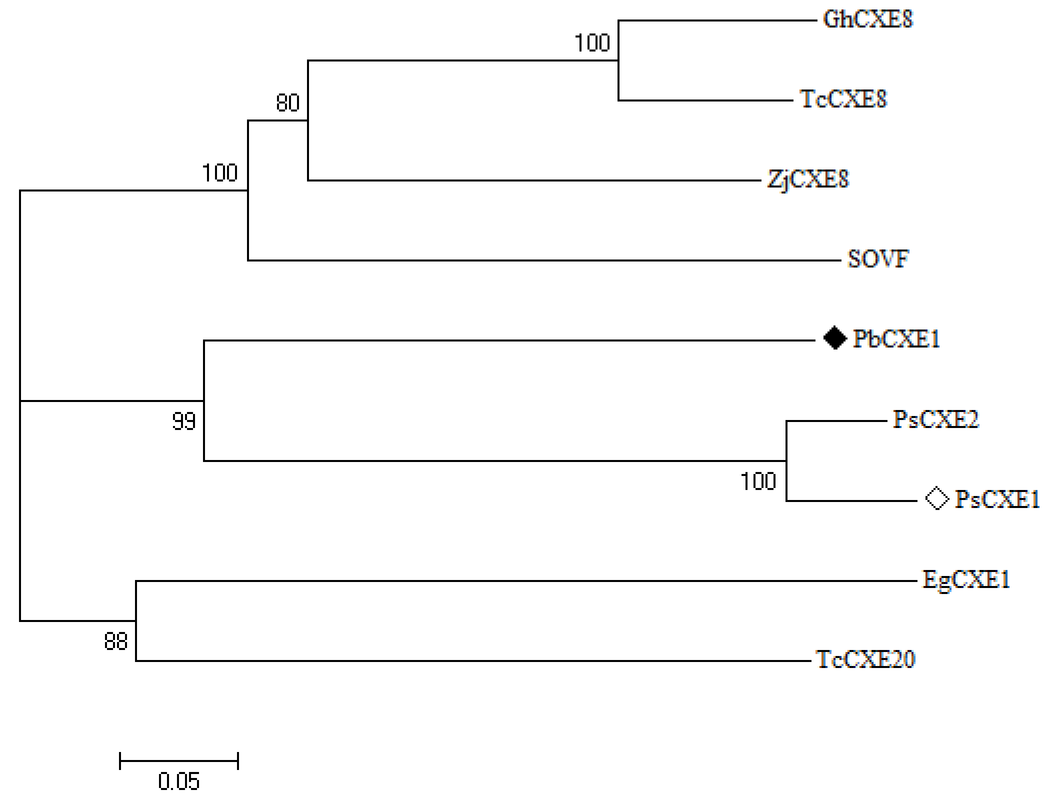

Fig.S5-E2. Unrooted neighbor-joining phylogenetic tree for CXE1 selected consensus sequence exploiting RNA-seq data obtained from *Papaver bracteatum* genus from a BLASTP search of the NCBI non-redundant protein database, constructed using MEGA 7 software. Bootstrap frequencies for each clade were based on 1,000 iterations.

Abbreviated species names are given before gene identifiers for each protein related to CXE1 are as follows:

PsCXE2, *P. somniferum* carboxylesterase 2 (AIY34373.1); PsCXE1, *P. somniferum* carboxylesterase 1 (AFB74618.1); PbCXE1, *P. bracteatum* carboxylesterase 1; SoSOVF, *Spinacia oleracea* SOVF_070390 isoform A (KNA18479.1); GhCXE8, *Gossypium hirsutum* carboxylesterase 8 (XP_016748284.1); TcCXE, *Theobroma cacao* carboxylesterase 8 (XP_017969789.1); ZJCXE8, *Ziziphus jujuba* carboxylesterase 8 (XP_015893007.1); EgCXE1, *Eucalyptus grandis* carboxylesterase 1-like (XP_010061765.1); TcCXE20, *T. cacao* Carboxyesterase 20, putative (EOY13805.1)

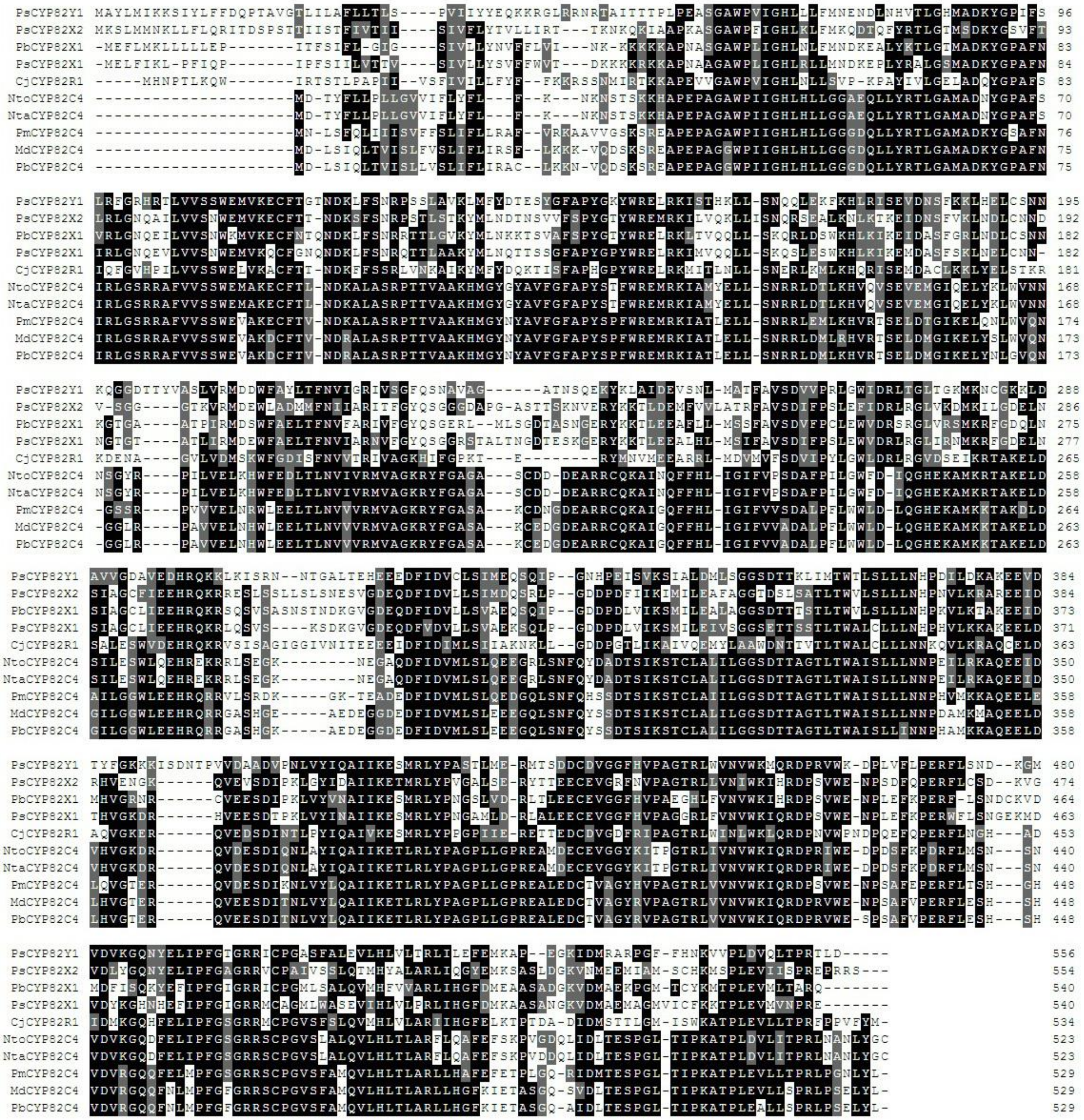

Fig.S5-F1. Sequence alignment of the amino acid sequence of identified CYP82X1 from *P. bracteatum* and its homologues in *P. somniferum*. Residues highlighted in black are identical and those in dark gray are similar. Percentage of sequences which must agree for identity or similarity coloring to be added 50%. Hyphens represent gaps inserted for optimal alignment.

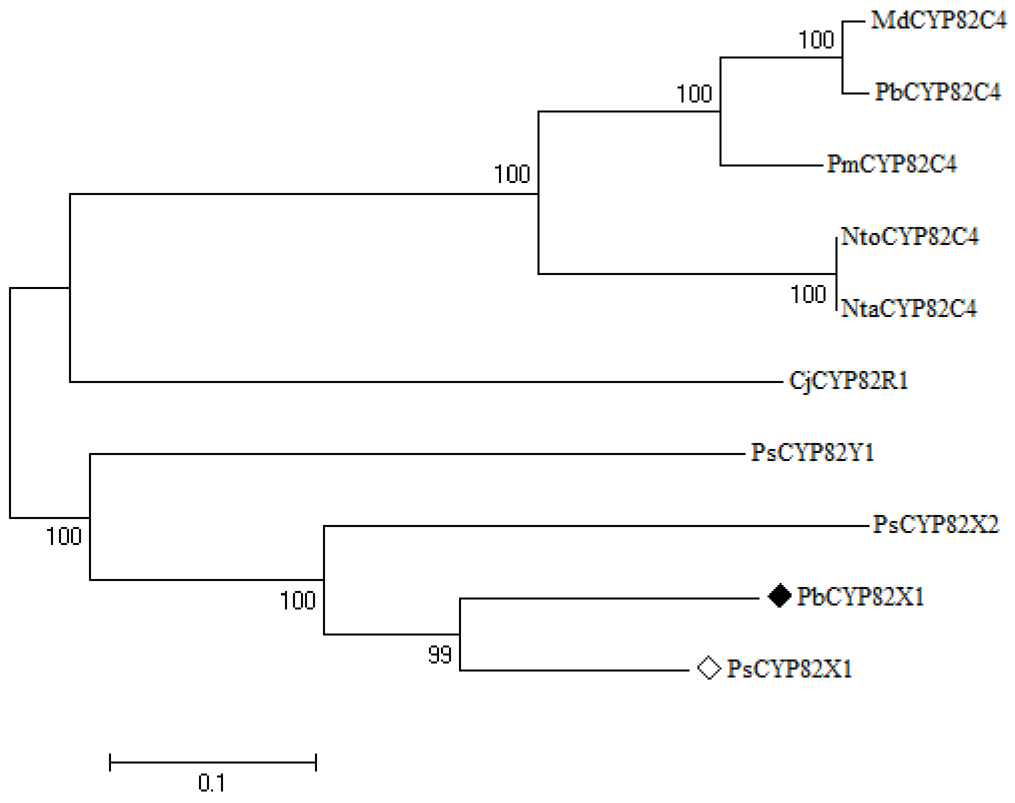

Fig.S5-F2. Unrooted neighbor-joining phylogenetic tree for CYP82X1 selected consensus sequence exploiting RNA-seq data obtained from *Papaver bracteatum* genus from a BLASTP search of the NCBI non-redundant protein database, constructed using MEGA 7 software. Bootstrap frequencies for each clade were based on 1,000 iterations. Abbreviated species names are given before gene identifiers for each protein related to CYP82X1 are as follows:

PbCYP82X1, *P. bracteatum* CYP82X1; PsCYP82X1, CYP82X1 *P. somniferum* (AFB74614); PsCYP82X2, *P. somniferum* CYP82X2 (AFB74616); PsCYP82Y1, *P. somniferum* CYP82Y1 (AFB74617); CjCYP82R1, *Coptis japonica* var. dissecta CYP82R1 (BAF98472.1), NtCYP82C4, *N. tomentosiformis* CYP82C4 (XP_009599169); NtCYP82C4, *N. tabacum* CYP82C4 (XP_016458765); PmCYP82C4, *Prunus mume* CYP82C4 (XP_016646999); MdCYP82C4 *Malus domestica* CYP82C4 (XP_008378409); PbCYP82C4, *Pyrus x bretschneideri* CYP82C4 (XP_009364162)

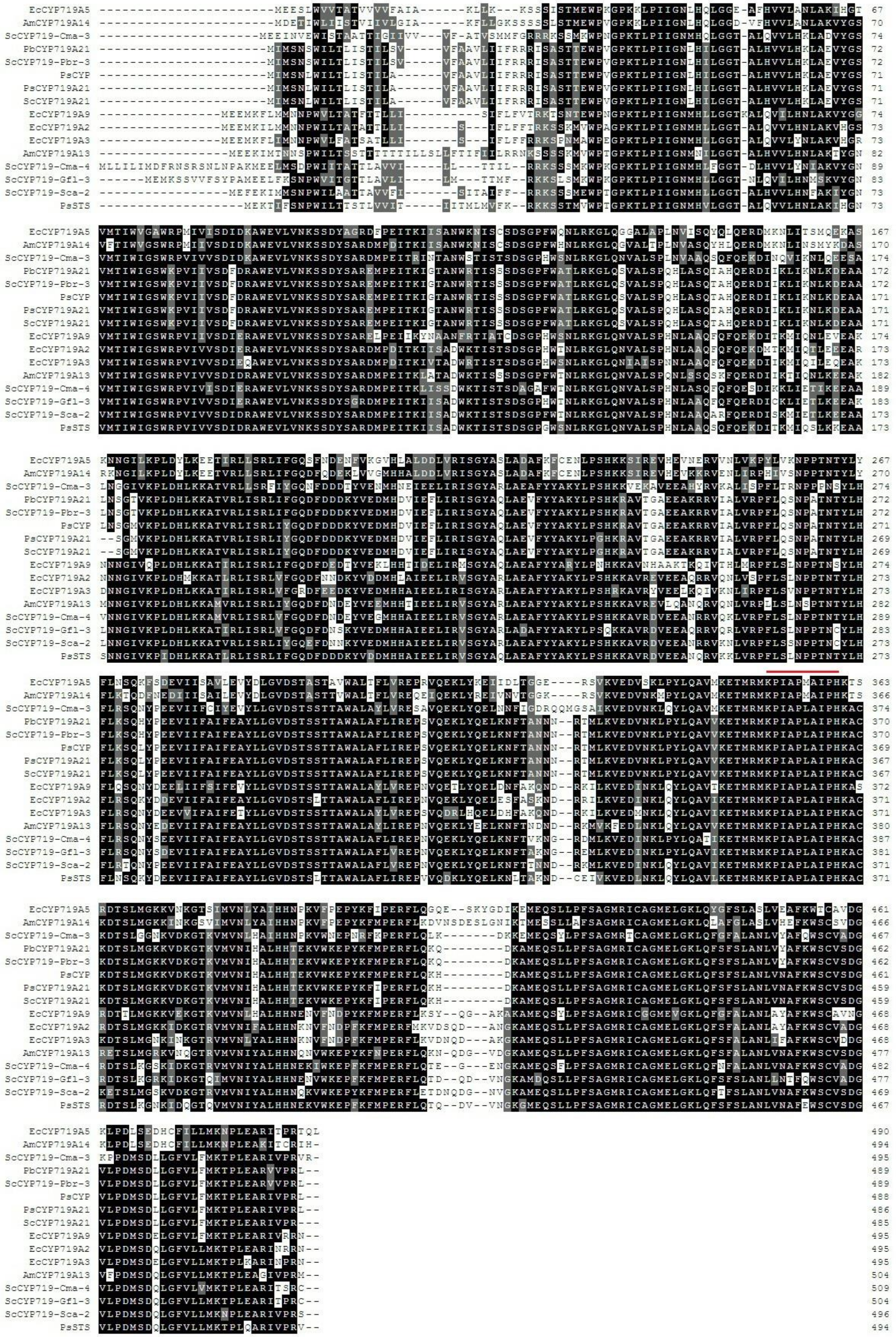
EcCYP719A5
AmCYP719A14
ScCYP719-Cma-3
PbCYP719A21
ScCYP719-Pbr-3
PsCYP
PsCYP719A21
ScCYP719A21
EcCYP719A9
EcCYP719A2
EcCYP719A3
AmCYP719A13
ScCYP719-Cma-4
ScCYP719-Gfl-3
ScCYP719-Sca-2
PsSTS

Fig.S5-G1. Sequence alignment of the amino acid sequence of identified AT1 from *P. bracteatum* . Residues highlighted in black are identical and those in dark gray are similar. Percentage of sequences which must agree for identity or similarity coloring to be added 50%. Hyphens represent gaps inserted for optimal alignment. The red line above the alignment indicates the position of the conserved KPIAPXXXPH motif among CYP719s.

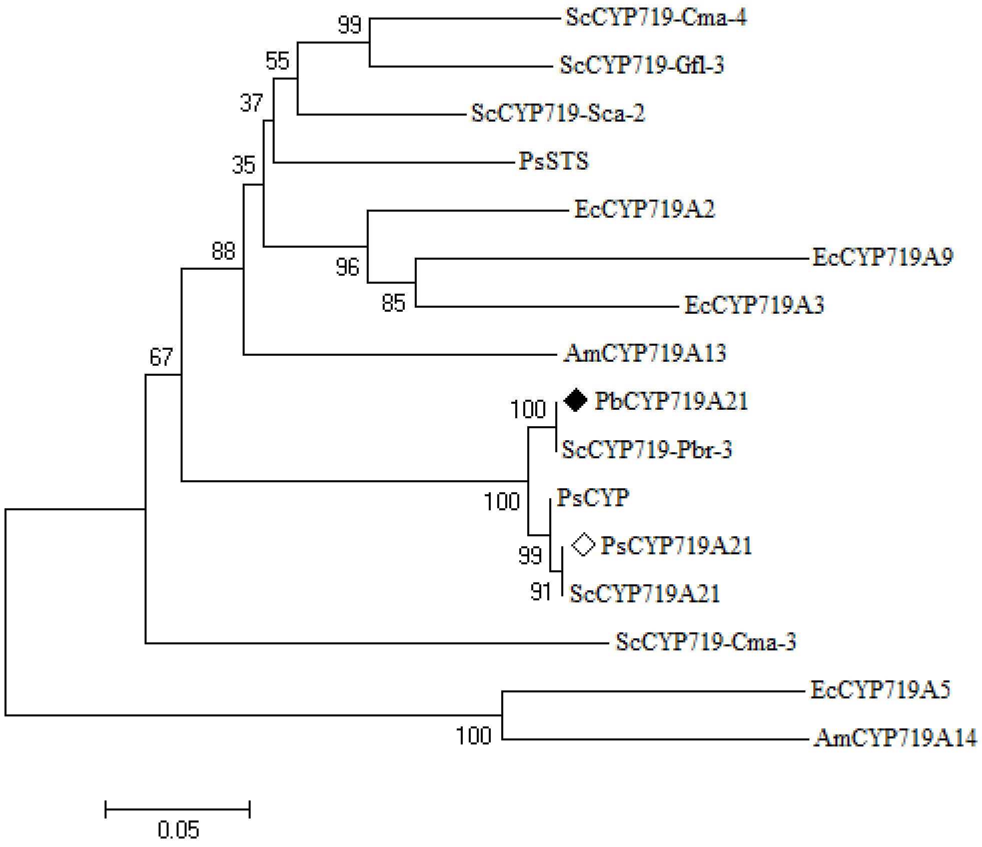

Fig.S5-G2. Unrooted neighbor-joining phylogenetic tree for CYP719A21 selected consensus sequence exploiting RNA-seq data obtained from *Papaver bracteatum* genus from a BLASTP search of the NCBI non-redundant protein database, constructed using MEGA 7 software. Bootstrap frequencies for each clade were based on 1,000 iterations. Abbreviated species names are given before gene identifiers for each protein related to CYP719A21 are as follows:

PsCYP719A21, *P. somniferum* CYP719A21 (AFB74615); PbCYP719A21, *P. bracteatum* CYP719; ScCYP719, synthetic construct CYP719 (ANY58171); EcCYP719A2, *Eschscholzia*

californica stylopine synthase (BAD98250.1); CYP719A3, *E. californica* stylopine synthase (BAD98249.1); ScCYP719A21, synthetic construct CYP719A21 (ANY58174); ScCYP719-Cma-4, synthetic construct CYP719-Cma-4 putative CYP719 (ANY58150); CYP719A5, *E. californica* cheilanthifoline synthase (BAG75113.1); CYP719A9, *E. californica* (BAG75114.1); CYP719A13, *A. mexicana* stylopine synthase (ABR14721.1); CYP719A14, *A. mexicana* cheilanthifoline synthase (ABR14722.1); PsCYP, *P. somniferum* cytochrome P450, partial (AFI57927); ScCYP719-Sca-2, synthetic construct putative CYP719-Sca-2 (ANY58180); ScCYP719-Gfl-3, synthetic construct putative CYP719 (ANY58160); PsSTS, *P. somniferum* stylopine synthase (ADB89214); ScCYP719-Cma-3, synthetic construct putative CYP719 (ANY58149)

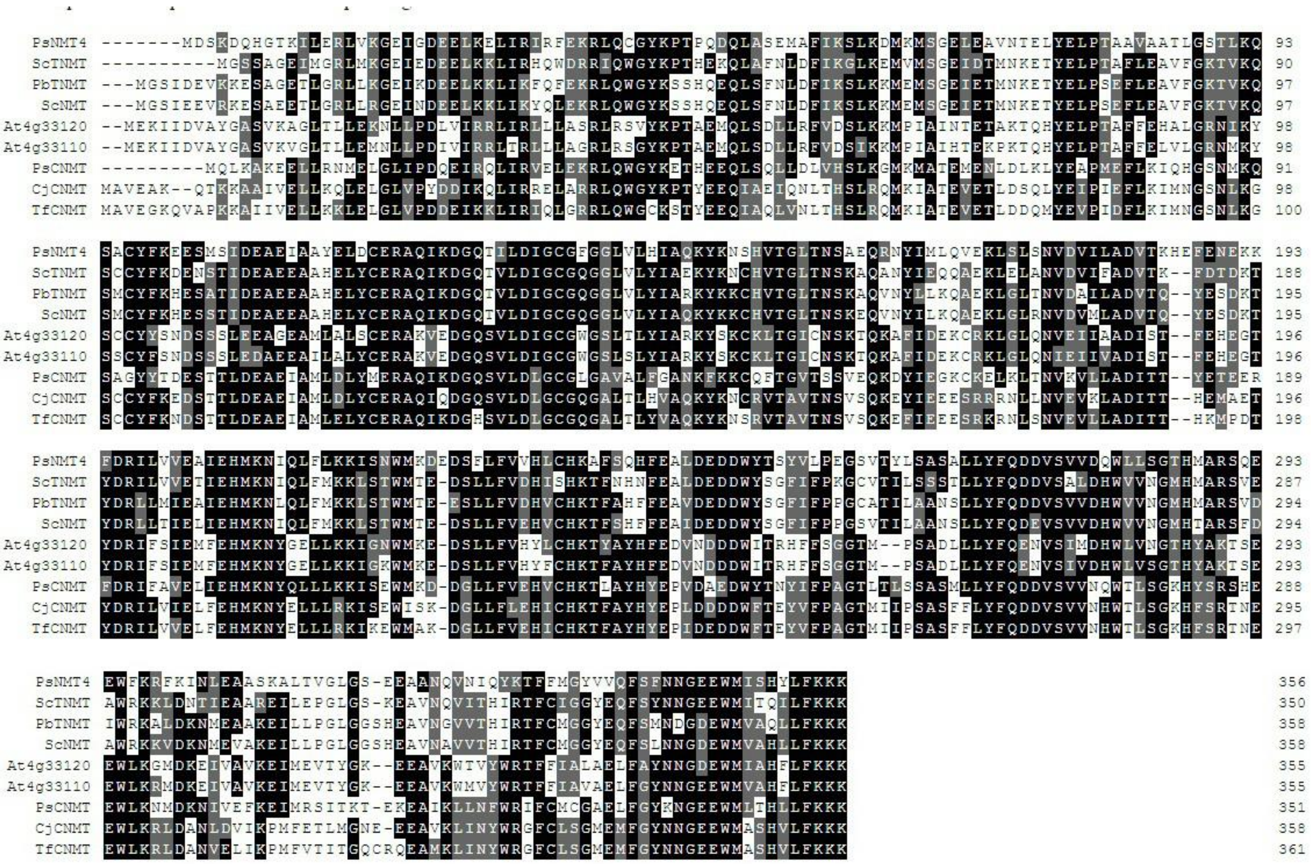

Fig.S5-H1. Sequence alignment of the amino acid sequence of identified TNMT from *P. bracteatum*. Residues highlighted in black are identical and those in dark gray are similar. Percentage of sequences which must agree for identity or similarity coloring to be added 50%. Hyphens represent gaps inserted for optimal alignment.

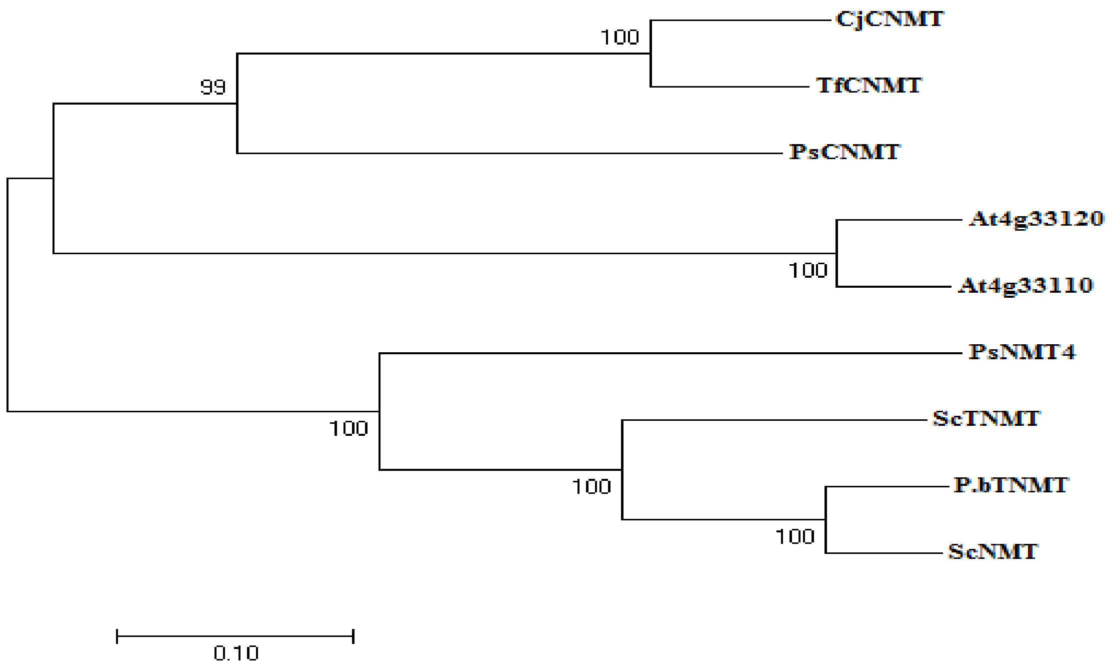

Fig.S5-H2. Unrooted neighbor-joining phylogenetic tree for TNMT selected consensus sequence exploiting RNA-seq data obtained from *Papaver bracteatum* genus from a BLASTP search of the NCBI non-redundant protein database, constructed using MEGA 7 software. Bootstrap frequencies for each clade were based on 1,000 iterations. Abbreviated species names are given before gene identifiers for each protein related to TNMT are as follows:

PbTNMT, *P. bracteatum* tetrahydroprotoberberine N-methyltransferase (ACO90237.1); NMT, N-methyltransferase synthetic construct (ANY58200); TNMT, tetrahydroprotoberberine N-methyltransferase synthetic construct (ANY58195); PsNMT4, *P. somniferum* N-methyltransferase (AOR51553); CjCNMT, *Coptis japonica* coclaurine N-methyltransferase (BAB71802); PsCNMT, *P. somniferum* S-adenosyl-L-methionine:coclaurine N-methyltransferase (AAP45316); TfCNMT, *Thalictrum flavum* (S)-coclaurine N-methyltransferase (AAU20766); At4g33120, *A. thaliana* At4g33120 ORF (NP_195038); At4g33110, *A. thaliana* At4g33110 ORF (AAM65762)

Supplementary Information 7

S7. The illustration of alignment of CYP82Y1 promoter region in Ps#7 and HN1 variety. The insertion has been accrued in, approximately, 400 base pair upstream of Translation Start Site (TSS). The ATG codon is highlighted in yellow, and blue sequences indicate the part of first exon.

```
Ps#7   GTAACGGAAATGAAACAACACTACCCAAATACTTATAAAGATGGGACTGTAAAGCTATTG  60
HN1    GTAACGGAAATGAAACAACACTACCCAAACACTTATAAAGATGGGACTGTAAAGCTATTG  60
       ***************************** ******************************

Ps#7   AGAGATTTCATGTTCAAAATTTGAGAAGAAATGCACACTTCTGAAACAGAAAGTATGAGA  120
HN1    AGAGATTTCATGTTCAAAATTTGAGAAGAAATGCACACTTCTGAAACAGAAAGTATGAAA  120
       ********************************************************** **

Ps#7   AGTCGGCAGGATCGACATTGTTGGAAGGTAACGACCACAAATAGCCGATAGGATAGTAAA  180
HN1    AGTCGGCAGGGTCGACATTATTGGAAGATAATGACCACAAATAGCCGATAGGATAGTAAA  180
       ************** *********** ***************** **************************************

Ps#7   AAATCATTATGTCAACTATATGATTTTTGACCTACATGAAAGGTGTTACAAATGCACTAT    240
HN1    AAATCATTATGTCAACTATATGATTTTTGACCTACATGAAAGGTGTTACAAATGCACGAT    240
       ********************************************************* **

Ps#7   TATTAGTCGGCAATATATTAATTTCATAACATGTCGAATTATAGTCGTCAAGGTTGAGAA   300
HN1    TATTAGTCGGCAATATATTAATTTCATAACATGTCGAATTGTAGTCATCAAGGTTGAGAA   300
       **************************************** ******  ******************

Ps#7   TTGTAAACATGAACGACTAAAAATCTTATTAATCTCAAAACCCGATGATCCAAATTTTCA  360
HN1    TTGTAAACATGAACGACTAAAAATCTTATTAATCTCAAAACCCGATGATCCAAATTTTCA  360
       ************************************************************

Ps#7   ATTTTTTCTTGATTTTTATTGTTCCGTTTTGATGCTTCGCTTTTGTTTTTGTTTTTCTTT    420
HN1    ATTTTTTCTTGATTTTTATTTTTCCGTTTTGATGCTTCGCTTTTGTTTTTGTTTTTCTTT    420
       ********************  *************************************

Ps#7   TTTGATTTCATCATGTATAGTTATAGTCTTGAAGAAAGATTTGAAAATTGTTTAGATAGG  480
HN1    TTTGATTTCATCATGTATAGTTATAATCTTGAAGAAAGATTTGAAAATTGTTTAGATAGG  480

Ps#7   TTTTAGTCGGTATG--GTTG--------TTTTTTTGGAACCAATTGATCTTTAAATATCT   530
HN1    TTTTAGTCGGTATGTTGTTGTTTTTTTTTTTTTTTTGGACAATTTGATTTTTTAATACCT   540
       ******************     ******          ********* *  * *  **** *****  *********** **

Ps#7   CAAAAACACCCTTAAGAGCAACTGCATGGACGACTAAATTCAAATTTGAATGTCGA       586
HN1    TTAAAACACCC---------------------------------------------
```

```
Ps#7   AAGACCAATTCCAAATATAGTCGGGCGTTTATAATAATGTCCGCCTACCCGACGGG   642
HN1    ---------------------------------------------------------

Ps#7   CGTTGGTAAAATGTCCGTCTGTAGCCGCGCGTTCGTAAAGTTAACGCCTGATGCAGG   699
HN1    ---------------------------------------------------------

Ps#7   GCGTTGGTATAATGTCCGCCTGGATGGGGCGTTGGTATAATGTCCGCCTGAATGGG   755
HN1    --------------------------------------------------------

Ps#7   GCATTGGTATAATCAAACGCCTGGCATGGGGCGTTGGTATAATGTCCGCATGGATGG   812
HN1    ---------------------------------------------------------

Ps#7   GGCGTACGTAAAGTTAACGCCGGACACCCAGGCGTTGATATAGTCATGATCCCAAATTT   871
HN1    -----------------------------------------------------------

Ps#7   GAAAAGTTTTGAAAACTTTACTTGGGGCGTTGTCTTTATCAACGCCCCATTTTTTTTTC   931
HN1    ------------------------------------------------------------

Ps#7   TTGAACCCGGGCGAACGTATAATCTCCGTCCCACACACCAGGCGTTGCTATAGTTCAC   989
HN1    ----------------------------------------------------------

Ps#7   GCCCCATACAGGCGTTATCTTTATCAACGCCCCATACCAGGCGTTATCTTTATCAACGC   1048
HN1    -----------------------------------------------------------

Ps#7   CCCATGCCAGGCGTAATCTTTATCTACGCTGGACGATGAAACGGACTTTATATCAAC   1105
HN1    ------------------------------------AAA------------------
                                           ***
```

```
Ps#7  GCGCGACCAAATTTACTCGTCACCCGCTACGCCACAGGACGGACTAAGCCCAAATTT   1162
HN1   - - - - - - - - - - - - - - - - - - CACCC - - - - - - - - - - - - - - - - - - - - - - - - - - - - - - -
                                          *****

Ps#7  AATTTTTTTTTTAGTCTTTGGTATTTAGTTATGCTGGCACCACTGTGAACGAT   1215
HN1   - - - - - - - - - - - - - - - - - - - - - - - - - - - - - - - - - - - - - - - - - - - - - - - - - - -


Ps#7  CTTAGCCCACTAGTTTGAATTGATATAACCCACTTAATGTTGGTATACCCAATTTAGCAA   1355
HN1   CTTAGCCCACTAACTTGAATTGATATAACCCACTTAATGTTGGTATACCCAATTTAGCAA   611
      ******************   **********************************************************

Ps#7  GGTTTTCCTTTTCAATTTTTTTTTTTGCCCTTTTCAAAAAAGTTCAGAAAAACATTATTGA   1415
HN1   GGTTTTCCTTTTCAATTTGTTTTTTGCCCTTTTCAAAAAAGTTCAGAAAAACATTATTGA   671
      ************************  **********************************************************

Ps#7  TGTGGACCAGTACCACTCCACTGGATTCCTCGAGAAAATTTTGACAAATA - - - - - - - - - - -   1465
HN1   TGTGGACCAGTACCACTCCACTGGATTCCTCGAGAAAATTTTGACAAATAATATATATAT   731
      ************************************************************************

Ps#7  - - - - - ATATATATATATATATAATATAAGCCATTAAACACTTATCTACAATTACAATAATT   1521
HN1   ATATATATATATATATATATAATATAAGCCATTAAACACTTATCTACAATTACAATAATT   791
              ******************************************************************************

Ps#7  GAGTAATTTCAGTTCATTCATGGCGTATTTGATGATCAAGAAATCTATCTATTTGTTTTT   1581
HN1   GAGTAATTTCAGTTCATTCATGGCGTATTTGATGATCAAGAAATCTATCTATTTGTTTTT   851
      ********************************************************************************

Ps#7  TGATCAACCAACTGCAGTTGGCACTCTTATACTTGCTTTTCTGCTGACAC   1631
HN1   TGATCAACCAACTGCAGTTGGCACTCTTATACTTGCTTTTCTGCTGACAC   901
      ********************************************************************
```

Supplementary Information 8

S8. Putative identified genes (full CDS), involved in noscapine biosynthesis pathway, and protein sequences obtained from CDS prediction from papaver bracteatum

trnascriptome data which are accessible on Sequence Read Archive (SRA) database. These Reads are assembled with the “align-then assemble” strategy utilizing

Codon Code Aligner v. 5. 0.1. Program.

>Putative papaver bracteatum acetyltransferase1 (PbAT1) (FULL CDS)

ATGGAAGCTTTGTCTAGTGTTGATGTCGAAGTGATCTCCAAGGAAACAATTAAACCCACAACTCCAACCCCATATC
AACT

TAGAAACTTCAATCTGTCCCTTCTTGATCAATATTGTCCTATAGTTTATGTTCCAATCATTCTTTTCTACCCTGCTGCA
G

TCACTAATAGTTCTGGTAGTAAGCACCACGATGATCTTAACTTGCTTAAGAGTTCTCTTTCTGAAACGCTGGGTCAA
TTT

TATCCACTGGCCGGTAGGATGAAAGACAACATTGTAGTCGACTGTAACGATGAAGGTATCGACTTTATCGAAGTA
AAAAT

CAAGAGTAAATTGTGCGACTTCATGATGAATCCAGATGTACGCTTAAGTCTGCTTCTCCCATCTGGAGTCGTTTCCA
TGA

ACTTCGTTAAGGAAGCACAGGTGATTGTTCAAGTGAACATATTTGATTGCGGTGGAGCAGCCATTTGTTTGTGTAT
ATCA

CACAAGATTGCAGATGCATGCACCATGAGTACATTCACTCGTTATTGGGCAGCCACCACCAACACAGCTCGTCTTG
GGGG

AGCTATCGGTCCTCCAAACACAAATCCGTTGTTTCTTCCTTCTTTCGACTCGGCATCTCTTTTCCCACCTAACGAACA
AT

TGGCATCTCAATCAGCGATGCCATCCGTTACTTATCCTACTGAGGATAGCCGAAGACATATAGTTGTCAGCAAAAG
ATTT

GTGTTCGATGCGGTAAAGTTAAAATCTGTACGTGAAAAGCTACAAGTATTGATGCATGATAAATACAGATCCCGTA
AGCC

AACAAGGGTTGAGGTGGTTTCCGCTCTAATATGGAAGGCAGCCATGAAATCCGCTCCGTCTGGTGCTTCATCGAC
GGTAA

ACCATGCCGTGAACTTTAGAAAGAAAATGGACCCACCTTTACAAGATGTGTCATTCGGAAATCTTTGTCAGGTCGT
TGCA

GCATTATTACCAGCAACAACGCCGGCAACAACAGAAAATTCAGATAACAAGACAGTTAATAGTACAAGTAATGAA
GTGCA

AGTGGCACTTGATGAGTTAAATGATTTCGTAGCTCAATTGAGAGGCGAAATAGATAAGGTAAAGGGCGACAAAG
GTTGCA

TGGAGAGAATGTTTCTAAATTTCATGAATGGTTATGATGCCTCCGTAACGAAAGACAGTGATGTTGAAGATGAAG
TGATA

GCTTTTTGGATGACTAGCTGGTGCAACTTTGGTTTATATGATGCTGATTTCGGTTGGGGAAAGCCAATTTGGGTAA
CTAC

TGATCCATTTATCGAGCCGAACAAGAACATAATTTACATGATTGATACAAAATGTGGTGAAGGAATAGAAGTGTG
GGTAA

ACTTTCTTGAGGATGATATGGCTAAGTTCGAGCATCACCTAAGCGAGATCCTCGAATTGTTTTGA

>PbAT1 (Protein sequence)

MEALSSVDVEVISKETIKPTTPTPYQLRNFNLSLLDQYCPIVYVPIILFYPAAVTNSSGSKHHDDLNLLKSSLSETLGQF

YPLAGRMKDNIVVDCNDEGIDFIEVKIKSKLCDFMMNPDVRLSLLLPSGVVSMNFVKEAQVIVQVNIFDCGGAAICLCIS

HKIADACTMSTFTRYWAATTNTARLGGAIGPPNTNPLFLPSFDSASLFPPNEQLASQSAMPSVTYPTEDSRRHIVVSKRF

VFDAVKLKSVREKLQVLMHDKYRSRKPTRVEVVSALIWKAAMKSAPSGASSTVNHAVNFRKKMDPPLQDVSFGNLCQ
VVA

ALLPATTPATTENSDNKTVNSTSNEVQVALDELNDFVAQLRGEIDKVKGDKGCMERMFLNFMNGYDASVTKDSDVED
EVI

AFWMTSWCNFGLYDADFGWGKPIWVTTDPFIEPNKNIIYMIDTKCGEGIEVWVNFLEDDMAKFEHHLSEILELF

>Putative papaver bracteatum carboxylesterase1 PbCXE1 (full CDS)

TAATTTTGAAGCTCCTGATAAAATGAAGTCTTTGACTCTTGGTAACATGATTTCAAGTTCTTCAGGCTCAAAGTAAA
CCA

TTCCATGATACCCGCCTTCTTGCATAAAAGTTTCAACCTTGACACCTTTTCCTTCCAACATCTTCACAAATTCAACTTG
TC

TATCAATCAATGGATCTCCATCACACCCAATAACCAAACATTTCTTTATAAACCCACATTTCTTTCGTAACGATTCGT
CG

TCTTCGTTGATCAAAAGATTACAATAAGGATGATTTCTATTAGACCCAATCGGCAAAGACAATTCCCACATCAAGTC
AC

TTACCGGGAGAGACAAAATCTTGTCATTGATTAGTCTCAGTTCAGAACTAGTCCTTTCAATTCCGCCCAGAAATGGT
TGATT

CAATATAACCCCGGAAATTTTCAAAGGGCTAAGATCCAATTCCGATGCTCTTAAAGAAACGTTGTAAGCAATATTTC
CACCT

GCACTTTCTCCCATAATATAACAATTTGTAAAATCAGCGTAATCCCTTAGCCACGGCTCACTTGGTTTACCTCCGGA
AGCTT

GATGTTTGACCCAGTTTAGAGCATCAACAGCATCATCATAAGCAGCCGGTAATCGACTTTCCGGTGCAAGACGATA
CTC

TACTGAAAGTACTATTGCCGGCAAAGCGTTTGCCTTGGATTTGCAGTAATCATGATATATAGTCCAAAATGTGCTG
CAAAG

AATGAACCCACCACCATGGTAATAAATTATGACCGGAAGCTTATTTGCTGTGGGTAAATCTTGAATTGTGGGTTTG
AAGATA

CGAATCCAAGTATTGTGTTGAGCATTGAGAAGTATGTCTTTGGAATTTGGATCATCTGTCGTGTTTGTAATTTGAAG
TGGG

AGATTTCTTGTCAGGGTATCAAGATCAGGATCGTGAACAACCATTAAGGCTTCGTAAGGATCGATGGTACTACTAG
TAGTAG

TTGAATGTTGATCTGCCAT

>PbCXE1 (Protein sequence)

MADQHSTTTSSTIDPYEALMVVHDPDLDTLTRNLPLQITNTTDDPNSKDILLNAQHNTWIRIFKPTIQDLPTANKLPVIIY
YHG

GGFILCSTFWTIYHDYCKSKANALPAIVLSVEYRLAPESRLPAAYDDAVDALNWVKHQASGGKPSEPWLRDYADFTNCY
IMGES

AGGNIAYNVSLRASELDLSPLKISGVILNQPFLGGIERTSSELRLINDKILSLPVSDLMWELSLPIGSNRNHPYCNLLINEDD
E

SLRKKCGFIKKCLVIGCDGDPLIDRQVEFVKMLEGKGVKVETFMQEGGYHGMVYFEPEELEIMLPRVKDFILSGASKL

>Putative papaver bracteatum cytochrome p450 CYP82X1 (PbCYP82X1) (full CDS)

ATGGAGTTCTTGATGAAGTTATTATTGTTACTTGAACCAATCACTTTTAGTATTTTTCTTGGTATAGGTTCTATTGTT
CTTCTAT

ACAATGTCTTCTTCTTGGTTATTAACAAGAAAAAGAAGAAGAAAGCACCAAATGCATCAGGGGCATGGCCGTTGA
TAGGCCATCT

CAATCTTTTCATGAATGATAAGGAAGCGTTGTATAAAACACTAGGAACCATGGCCGACAAGTACGGACCTGCATTC
AACGTTCGA

TTAGGCAACCAAGAAATCCTTGTGGTGAGTAATTGGAAGATGGTAAAAGAATGTTTTAACACTCAAAATGATAAG
CTATTTTCGA

ATCGTCGAACTACATTGGGTGTGAAATACATGCTTAACAAGAAGACCTCTGTTGCCTTTTCACCATATGGAACATAT
TGGAGGGA

GCTACGAAAGCTAACGGTGCAACAATTGCTCTCTAAGCAACGTTTAGATTCGTGGAAACATTTGAAAATCAAAGAA
ATAGATGCT

TCATTTGGTAGACTTAACGATTTATGCAGCAACAACAAGGGTACTGGAGCAGCTACCCCAATTAGGATGGACAGTT
GGTTTGCCG

AGTTGACGTTCAACGTGTTCGCAAGAATTGTCTTTGGCTACCAAAGTGGAGAAAGGTTGATGCTATCAGGTGATAC
GGCATCCAA

CGGGGAGAGGTACAAGAAAACATTAGAAGAAGCATTTCTCCTTATGTCAAGCTTTGCGGTTTCTGACGTATTCCCA
TGCTTAGAG

TGGGTAGACAGATCAAGAGGCCTTGTAAGGAGCATGAAACGCTTTGGAGATCAGCTAAATTCAATTGCAGGGTGT
CTTATTGAGG

AGCATCGCCAAAAGAGATCACAATCCGTATCAGCATCAAATTCTACAAATGATAAAGGAGTTGGTGATGAACAAG
ACTTCATTGA

TGTTCTCTTATCGGTTGCTGAACAATCACAAATTCCTGGAGATGACCCTGATTTGGTCATCAAGTCTATGATTTTGG
AAGCCTTA

GCAGGTGGGAGTGACACAACAACATCAACCCTAACTTGGGTTCTTTCACTGCTATTGAACCACCCCAAAGTGTTAA
AGACGGCAA

AAGAGGAAATAGATATGCACGTCGGACGTAATCGATGCGTAGAAGAGTCAGATATTCCCAAGCTCGTTTATGTCA
ATGCAATTAT

CAAAGAATCAATGAGATTGTATCCAAATGGGTCATTGGTTGATCGATTGACGTTGGAAGAGTGTGAAGTTGGTGG
ATTCCATGTC

CCAGCTGAGGGACACTTATTCGTAAACGTTTGGAAGATCCATAGAGATCCGAGTGTGTGGGAGAATCCTCTGGAG
TTCAAGCCAG

AGAGATTTTTGAGTAATGATTGCAAGGTGGATATGGATTTCATAAGTCAAAAATATGAATTCATACCATTCGGGAT
AGGTCGGAG

GATATGTCCTGGTATGCTTTCAGCATTACAGGTGATGCATTTTGTGGTAGCCCGTCTTATTCATGGGTTTGATATGG
AAGCAGCA

AGTGCCGATGGGAAAGTGGATATGGCAGAAAAGCCAGGCATGACTTGCTATAAGATGACACCTCTTGAAGTTATG
CTCACGGCTC

GACAGTAG

>PbCYP82X1 (Protein sequence)

MEFLMKLLLLLEPITFSIFLGIGSIVLLYNVFFLVINKKKKKKAPNASGAWPLIGHLNLFMNDKEALYKTLGTMADKYGPAFNVRLGNQE

ILVVSNWKMVKECFNTQNDKLFSNRRTTLGVKYMLNKKTSVAFSPYGTYWRELRKLTVQQLLSKQRLDSWKHLKIKEIDASFGRLNDLCS

NNKGTGAATPIRMDSWFAELTFNVFARIVFGYQSGERLMLSGDTASNGERYKKTLEEAFLLMSSFAVSDVFPCLEWVDRSRGLVRSMKRF

GDQLNSIAGCLIEEHRQKRSQSVSASNSTNDKGVGDEQDFIDVLLSVAEQSQIPGDDPDLVIKSMILEALAGGSDTTTSTLTWVLSLLLN

HPKVLKTAKEEIDMHVGRNRCVEESDIPKLVYVNAIIKESMRLYPNGSLVDRLTLEECEVGGFHVPAEGHLFVNVWKIHRDPSVWENPLEF

KPERFLSNDCKVDMDFISQKYEFIPFGIGRRICPGMLSALQVMHFVVARLIHGFDMEAASADGKVDMAEKPGMTCYKMTPLEVMLTARQ

>Putative papaver bracteatum cytochrome p450 CYP82Y1 (PbCYP82Y1) (FULL CDS)

ATCTAGTGTGCGTGGGGTGAGTAGAACATCTAGTGGCACTACCTTGTTGTTGAAAAAACCTGGTCTTGCCCTCATGTCAATTTCCCCCTCTGGTG

CCTTCATCTCGAACTCAAGAATAAGACGAGTGAGGACCAAATGCAAGACTTCTAAGGCAAAAGATGCACCGGGACATATCCGCCTGCCTGTTCCGA

ATGGTATCAGTTCATAATTCTGACCCTTCACATCTACCATCCCTTTGTCATTGCTCAAGAATCTCTCAGGTCGAAATACCAATGGATCATTCCAAA

CCCTTGGGTCTCGTTGCATCTTCCATACGTTAACCCATAATCTCGTCCCAGCTGGAACGTGGAAGCCACCAACCTCGCAATCATCACTGGTCATCCG

CTCCATTATTGTGCTGGCGGGGTATAATCGCATTGATTCTTTGATGATAGCTTGGATGTAGACGAGGTTAGGAACATCAGAAGCATCAACGACAGCC

ATCTTTTTCTTCCTGAAGTATGTATCCACTTCTTCTTTAGCTTTGTCCAATATGTCTGGATGGTTCAACAGCAAAGAAAGGGTCCATGTCATTATCA

ACTTTGTAGTGTCACTCCCACCCGATAACATGTCCAAGGCAATAGATTTGACAGAGATTTCGGGGTTGTTTCCCGGAATCTGTGATTGCTCCATAAT

CGACAAGCAAACATCGATGAAGTCTTCTTCTTCATGCTCCGTAAGTTCTCCTGCGGTATTTCTAGAAATTTTGAGTTTCTTTTGGCGATGAACCTCC

ACTGCATCCCCAACTACTGCATCTAATTTCTTTCCGCATTTCTTCATCTTTCCTGTAAGACCAGTCAATCGGTCAATCCACCCAAGGCATGGAACCA

CATCTGAAACGGCAAATGTTGCCATAAGATTTGACACTTCATCGATTGCAAGCTTGTATTTTTCCTGGCTGCTTGTGGCACCTGACACTGCATTTGA

TTGGAATCCGCTGACAATCCGTCCTATTACGTTGAATGTCAAGTAAGCGAACCAATCATCCATTCTCACAAGATTAG CCACAGATGTAGTTTCACCT

CCCTGTTTGTTGTTGCTGCATAACTCATGAAGCCTTTTAAAGGAGTTGTCCACTTCAGAAATCCGCAAGTGTTTGAA CTTCTCTAATTGCTGATTAG

AGAGGAGTTTATGTGTAGATATCTTCCGCAACTCTCTCCAGTACTTCCCATAAGGTGCAAAACCATAAGACTCAGT GTCATAAAACATAAGTTTAAC

CGCCAAGGAGGAAGGACGATTTGAAAAAAACTTGTCATTGGCACCTGTAAAACACTCCTTTACCATCTCCCAACTA CTAACAACTAGAGTTCTATGA

CTACCGAATCTTAAGCTGAAAATAGGTCCATATTTATCAGCCATGTTACCGAGAGTTGCATGATTTAAATCGTTTTC GTTCATGAAAAGAAGAAGAT

GACCTATCACTGGCCACGCACCTGATGCCTCTGGTAACGGAGTCGTTGTAATTGCGGTACATGACGATGAGGTCC GATTTCGCCTCAAACCCCTCTT

CTTCTGTTCATAGTAAATAATAACAGGTGAAAGTGTCAGTAGAAAAGCAAGTACTATAAGAGTGGAAACTGAAGT TGGTTGATCGGAAAACAAGTGG

AAAGATTTCTTGA

>PbCYP82Y1 (Protein sequence)

MAYLMIKKSFHLFSDQPTSVSTLIVLAFLLTLSPVIIYYEQKKRGLRRNRTSSSCTAITTTPLPEASGAWPVIGHLLLFMNEN DLNHATLGNMADKYG

PIFSLRFGSHRTLVVSSWEMVKECFTGANDKFFSNRPSSLAVKLMFYDTESYGFAPYGKYWRELRKISTHKLLSNQQLEK FKHLRISEVDNSFKRLHE

LCSNNKQGGETTSVANLVRMDDWFAYLTFNVIGRIVSGFQSNAVSGATSSQEKYKLAIDEVSNLMATFAVSDVVPCLG WIDRLTGLTGKMKKCGKKLD

AVVGDAVEVHRQKKLKISRNTAGELTEHEEEDFIDVCLSIMEQSQIPGNNPEISVKSIALDMLSGGSDTTKLIMTWTLSLL LNHPDILDKAKEEVDTY

FRKKKMAVVDASDVPNLVYIQAIIKESMRLYPASTIMERMTSDDCEVGGFHVPAGTRLWVNVWKMQRDPRVWNDP LVFRPERFLSNDKGMVDVKGQNY

ELIPFGTGRRICPGASFALEVLHLVLTRLILEFEMKAPEGEIDMRARPGFFNNKVVPLDVLLTPRTLD

>Putative papaver bracteatum cytochrome p450 PbCYP719A21 (PbCYP719A21) (FULL CDS)

ATGATCATGAGTAACTCTTGGATTCTTACGCTCATTTCTACCATATTATCAGTCGTCTTTGCTGCTGTGTTAATCATTT TCAGGAGAAGAATATCAGCATCCAC

AACGGAATGGCCTGTTGGCCCAAAAACATTACCGATCATAGGTAACTTGCACATTCTTGGAGGCACTGCTCTCCATGTCGTCTTACATAAACTAGCTGAAGTTT

ACGGCAGTGTAATGACGATATGGATTGGTAGTTGGAAACCTGTTATTATTGTTTCTGACTTTGATCGAGCTTGGGAAGTTCTTGTTAACAAATCTTCAGATTAT

TCAGCTCGTGAAATGCCTGAGATCACTAAAATCGGCACTGCAAATTGGAGAACAATTTCAAGTTCTGATTCTGGTCCATTTTGGGCCACTCTTCGTAAAGGTCT

TCAGAGTGTAGCATTATCCCCTCAGCATTTAGCATCGCAAACTGCACACCAAGAGAGAGATATAATAAAGTTGATCAAAAATTTGAAAGACGAAGCAGCTCTTA

ATTCTGGAACCGTTAAACCACTTGATCATCTCAAGAAAGCAACTGTTAGATTAATCAGTCGG-TTAATCTTTGGTCAGGATTTTGATGACGATAAGTATGTTGA

AGATATGCATGACGTGATCGAGTTTTTGATTCGTATTAGTGGTTATGCTCAACTTGCTGAGGTTTTCTACTATGCTAAATATCTACCAAGTCATAAGAGAGCTG

TAACTGGCGCCGAAGAAGCAAAAAGAAGAGTAATAGCTCTGGTGCGTCCTTTTCTTCAGTCAAACCCTGCTACGAACACTTACTTGCATTTTCTTAAATCGCAG

CACTACCCAGAAGAGGTTATCATATTCGCTATATTCGAAGCTTATCTATTAGGTGTTGATAGTACTTCTTCAACCACTGCATGGGCACTCGCATTCTTAATACG

TGAACCATCTGTTCAAGAGAAACTTTATCAAGAGCTTAAGAATTTCACAGCCAATAACAATCGCACAATGCTGAAAGTCGAAGATGTCAACAAATTACCATATT

TACAAGCTGTTGTTAAAGAAACAATGAGGATGAAACCAATTGCACCACTGGCAATTCCTCATAAAGCTTGTAAAGACACTTCATTGATGGGCAAGAAAGTTGAT

AAGGGAACTAAAGTTATGGTTAACATTCATGCTTTACATCATACTGAAAAAGTTTGGAAAGAACCTTACAAATTCATGCCAGAGAGGTTTCTGCAAAAACAAGA

TAAGGCGATGGAACAATCACTATTACCATTCAGTGCAGGTATGAGAATTTGTGCAGGAATGGAATTAGGAAAACTTCAATTTAGTTTTTCTCTTGCTAATCTTG

TATATGCTTTTAAATGGTCTTGTGTTTCTGATGGAGTGCTTCCTGACATGAGTGATTTACTGGGGTTTGTTCTGTTCATGAAAACCCCACTCGAAGCACGTGTA

GTTCCTCGTTTG

>PbCYP719A21 (Protein sequence)

MIMSNSWILTLISTILSVVFAAVLIIFRRRISASTTEWPVGPKTLPIIGNLHILGGTALHVVLHKLAEVYGSVMTIWIGSWKPVIIVSDFDRAWEVLVNK

SSDYSAREMPEITKIGTANWRTISSSDSGPFWATLRKGLQSVALSPQHLASQTAHQERDIIKLIKNLKDEAALNSGTVKPLDHLKKATVRLISRLIFGQDF

DDDKYVEDMHDVIEFLIRISGYAQLAEVFYYAKYLPSHKRAVTGAEEAKRRVIALVRPFLQSNPATNTYLHFLKSQHYPEE
VIIFAIFEAYLLGVDSTSS

TTAWALAFLIREPSVQEKLYQELKNFTANNNRTMLKVEDVNKLPYLQAVVKETMRMKPIAPLAIPHKACKDTSLMGKK
VDKGTKVMVNIHALHHTEKVWK

EPYKFMPERFLQKQDKAMEQSLLPFSAGMRICAGMELGKLQFSFSLANLVYAFKWSCVSDGVLPDMSDLLGFVLFMK
TPLEARVVPRL

>Putative papaver bracteatum O-methyltransferases (PbMT1) (FULL CDS)

ATGGCTACCAATGGCGAAATTTTCGATACCTATGGTCATAATCATCAAACAGCCACAGTCACTAAAATCACTGCTTC
TGATGACAGCAGTAATGATGTCTGTTA

TCTTTCAGAAACGGCTAACTTAGGGAAGTTAATATGCATTCCAATGGCATTAAGAGCTGCGATGGAGCTAAATGTG
TTCCAACTTATCTCAAAGTTTGGAACTG

ACGCAAAAGTTTCGGCTTCTGAAATTGCCTCTAAAATGCCAAACACGACGAGTAACCCAACAGCAGCTATCTATTT
AGATAGAGTTCTTCGACTGCTCGGTGCC

AGTTCTATTCTTTCCGTTTCTACCAGAAAAAAATTAATCAACGGAGGAGGAGAAGATAGAGTAGTACATGAGAAG
GTGTACGGGCTAACGAATTCGTCGTGCTG

TTTGGTCCCTCGAGAAGAAGACGGGGTGTCATTAGTCGAAGAATTGCTATTCACATCTGACAAGGTTGTTGTGGAT
AGTTTTTTCAAACTGAAATCTGTGGTGG

AAGAAAAAGACAGTGTGCCATTTGAGGTTGCTCATGGTGCTAAGATCTTTGAGTATGCTGCTACAGAACCAAGAA
TGAATAAGGTTTTTAACGATGGAATGGCA

GTTTTCTCTATTGTTGTTTTTGAAGCTGTTTTTAGAGTTTACGATGGATTTCTTGACATGAAAGAATTGTTAGATGTT
GGTGGTGGTATTGGTACTTCAGTTAG

TAAAATTGTTGCTAAATACCCTTTCATTCGCGGTGTCAACTTCGACTTGCCTCATGTTATTTCTGTTGCCCCTCAATA
CCCAGGGGTAGAGCATGTTGCAGGAG

ACATGTTCGAGGAAGTTCCAAAGGGTCAAAACATGTTGCTAAAATGGGTACTGCACGATTGGGGTGACGAACGAT
GTGTGAAGCTGTTAAAGAATTGTTGGAAG

TCATTACCTGCGGGTGGAAAAGTTTTGATAATCGAGTTTGTTCTTCCGAATGAACTTGGTAATAATGCCGAATCATT
CAATGCGTTGATTCCCGATTTACTCCT

GATGGCTCTGAATCCAGGCGGTAAAGAACGAACAATTTCGGAATATGATGATTTAGCCAAAGCAGCTGGATTTAT
AAAAACTATACCTATCCCTATCTCCAATG

GTCTTCATGTCATTGAGTTTCACAAA

PbMT1 (Protein sequence)

MATNGEIFDTYGHNHQTATVTKITASDDSSNDVCYLSETANLGKLICIPMALRAAMELNVFQLISKFGTDAKVSASEIAS KMPNTTSNPTAAIYLDRVLRL

LGASSILSVSTRKKLINGGGEDRVVHEKVYGLTNSSCCLVPREEDGVSLVEELLFTSDKVVVDSFFKLKSVVEEKDSVPFEV AHGAKIFEYAATEPRMNKV

FNDGMAVFSIVVFEAVFRVYDGFLDMKELLDVGGGIGTSVSKIVAKYPFIRGVNFDLPHVISVAPQYPGVEHVAGDMFE EVPKGQNMLLKWVLHDWGDERC

VKLLKNCWKSLPAGGKVLIIEFVLPNELGNNAESFNALIPDLLLMALNPGGKERTISEYDDLAKAAGFIKTIPIPISNGLHVI EFHK

Putative papaver bracteatum short-chain dehydrogenase/reductase (PbSDR1) (FULL CDS)

ATGGAAGGAGAACACATAAATGATCAGGGAGGAAATGGGAAGATAGTATGTGTAACAGGTGGTGCTGGATACTT GGCATCTTGGCTCATCTTGAGATTGCTTCAA

CGTGGTTACTCTGTTCGGACCACCGTTCGGTCCGACCCCAAATTTAAGGAAGATGTAAGCCACCTGAAAGCCCTTC CTGAGGCTGCCGAGAAGCTCCAAATTTTT

GAAGCAGATCTCGAAACCCCGGAAAGTTTTGATGCTGCAGTTGACGGATGTGTCGGTGTCTTTCTCGTTGCTCAAG GAATTGGTCTTGAACAAGCCTACACTCAA

GAAAAATTACTCAAGACATCCGTGGAAGGAACTCTTGGAATTCTCAAGTCATGCTTGAAGTCCAAAACAGTGAAA AAAGTTGTGTACACATCTTCTTCAGCTGCA

GCAATGATGGTAAGTAATCTCAAAGTTGTAACACAAATTGACGAGACAATGTGGTCAGAAGTTGACCATTTCATTG GCAAACCCGAACAAGTCATTCCTGCTGGT

CTCGCATATGTGGTTTCAAAGACACTGACAGAAAGAGCTGCCATGAAATTTTCTGACGAACATGGATTGGATCTTG TTACTATACTTCCATCTATGATTGTTGGA

CCATTTATCATCCCCAACCTTCCTGAGAGTGTTTCTCTGGCTCTTTCTGTAATTTTGGGTGATAGGACGAAGATGAT CCGTCTTAAACCTACAAATGCAGTACAC

ATAGATGATGTTGCTTCAGCACAAATATTTCTTTTTGAATGCCAAAATGCAAAAGGAAGACATATTTGTTCTTCAGT TGATTTTACAATACATGATGTGGCTAAA

TTTATAGCTGAAAAATATCCAGAATTTCAGTTACCAACTGATTTACTAAAGGAAATTGAGGAGGAAAAACCAGTTC ATCTATCCTCAGATAAGCTGTTGAGTATG

GGATTTCAGTTCAAATATAATTTTGAGGAGATGTTTGGTGATGCAATTCGAAGCTGCAAAGAGAAAGGTTTCCTT

PbSDR1 (Protein sequence)

MEGEHINDQGGNGKIVCVTGGAGYLASWLILRLLQRGYSVRTTVRSDPKFKEDVSHLKALPEAAEKLQIFEADLETPESF DAAVDGCVGVFLVAQGIGLEQAYTQE

KLLKTSVEGTLGILKSCLKSKTVKKVVYTSSSAAAMMVSNLKVVTQIDETMWSEVDHFIGKPEQVIPAGLAYVVSKTLTER AAMKFSDEHGLDLVTILPSMIVGPF

IIPNLPESVSLALSVILGDRTKMIRLKPTNAVHIDDVASAQIFLFECQNAKGRHICSSVDFTIHDVAKFIAEKYPEFQLPTDLL KEIEEEKPVHLSSDKLLSMGFQ

FKYNFEEMFGDAIRSCKEKGFL

Putative papaver bracteatum tetrahydroxyprotoberberine Nmethyltransferase (PbTNMT) (Full CDS)

ATGGGTTCAATAGATGAAGTGAAGAAGGAATCAGCAGGGGAAACACTTGGGAGATTGTTGAAAGGAGAGATTAA AGATGAGGAACTGAAGAAGCTGATCAAGTTTCA

GTTCGAGAAGCGTCTGCAATGGGGTTATAAATCATCTCATCAAGAACAACTCTCCTTCAACCTTGACTTCATTAAAT CCTTAAAAAAGATGGAAATGTCAGGAGAGA

TTGAGACAATGAACAAGGAAACTTATGAATTACCCTCAGAATTCTTAGAGGCAGTTTTTGGAAAAACAGTCAAGCA AAGTATGTGTTACTTCAAACATGAATCAGCA

ACAATAGACGAAGCAGAAGAAGCTGCACATGAATTATACTGTGAACGAGCACAGATCAAAGATGGACAAACTGTT CTCGACATTGGTTGTGGTCAAGGTGGTCTAGT

TCTATACATTGCTCGGAAATATAAGAAATGTCATGTTACAGGGCTCACTAATTCAAAGGCACAAGTCAATTACTTAC TCAAGCAAGCAGAGAAGCTTGGGCTGACAA

ATGTCGACGCCATACTAGCAGATGTCACTCAATATGAATCTGACAAGACATACGATCGACTACTTATGATTGAAGC CATAGAGCACATGAAAAACTTACAACTGTTC

ATGAAGAAACTATCAACTTGGATGACAGAAGAAAGTCTTCTCTTTGTGGACCATGTCTGCCATAAAACCTTTGCTC ACTTTTTTGAGGCAGTCGACGAGGATGACTG

GTACTCAGGTTTCATCTTCCCCCCAGGTTGTGCAACCATACTAGCTGCTAATAGTCTCCTCTATTTCCAGGATGATGT TTCAGTTGTGGATCATTGGGTTGTCAACG

GAATGCACATGGCTCGTTCAGTAGACATTTGGAGAAAGGCACTAGACAAAAATATGGAAGCTGCAAAAGAAATTC TATTACCTGGACTTGGAGGAAGCCACGAAGCA

GTAAATGGAGTCGTTACTCACATTAGAACATTCTGTATGGGAGGCTATGAACAATTCTCAATGAACGATGGAGAT GAGTGGATGGTTGCGCAGCTGCTTTTCAAGAA

GAAGTAG

PbTNMT (Protein sequence)

MGSIDEVKKESAGETLGRLLKGEIKDEELKKLIKFQFEKRLQWGYKSSHQEQLSFNLDFIKSLKKMEMSGEIETMNKETYE
LPSEFLEAVFGKTVKQSMCYFKHESAT

IDEAEEAAHELYCERAQIKDGQTVLDIGCGQGGLVLYIARKYKKCHVTGLTNSKAQVNYLLKQAEKLGLTNVDAILADVT
QYESDKTYDRLLMIEAIEHMKNLQLFMK

KLSTWMTEESLLFVDHVCHKTFAHFFEAVDEDDWYSGFIFPPGCATILAANSLLYFQDDVSVVDHWVVNGMHMARS
VDIWRKALDKNMEAAKEILLPGLGGSHEAVNG

VVTHIRTFCMGGYEQFSMNDGDEWMVAQLLFKKK

Supplementary Information 9

Table S9. The labels, origins and geographic coordinates of studied ecotypes of *P. somniferum* L in this research.

No.	Label	Origin	above mean sea level (meter)	Longitude (degrees minutes seconds)	Latitude (degrees minutes seconds)
1	Ps#1	Iran, Mazandaran, Yoush	2598.3	51° 42′ 6.286″	36° 10′ 19.720″
2	Ps#2	Iran, Hamedan, Nahavand	1529.0	48° 16′ 16.554″	34° 16′ 26.883″
3	Ps#3	Iran, Fars, Komareh sorkhi	1855.6	53° 17′ 11.486″	29° 26′ 9.231″
4	Ps#6	Iran, Zanjan, Abhar	1520.9	49° 14′ 14.023″	36° 8′ 43.922″
5	Ps#7	Iran, Hamedan, Nahavand	1643.0	48° 21′ 42.848″	34° 11′ 16.326″
6	Ps#8	Iran, Kermanshah	1357.7	47° 4′ 55.182″	34° 19′ 2.087″
7	Ps#9	Iran, Lorestan, Kohdasht	1186.9	47° 38′ 42.160″	33° 29′ 47.888″
8	Ps#10	Iran, Kurdistan, Sanandaj	1482.3	46° 59′ 58.551″	35° 18′ 17.763″
9	Ps#11	Iran, Lorestan, Aleshtar	1607.1	48° 13′ 48.239″	33° 53′ 31.094″
10	Ps#12	Iran, West Azarbaijan, Salmas	1367.4	44° 46′ 59.181″	38° 11′ 14.514″
11	Ps#14	Iran, Kerman	1745.4	56° 58′ 50.436″	30° 21′ 17.965″
12	Ps#16	Iran, Hamedan, Nahavand	1593.5	48° 20′ 23.746″	34° 12′ 13.484″

Supplementary Information 10

S10. HPLC chromatograms of noscapine extracted from *Papaver somniferum*. L. The standard sample (noscapine) at 50 ug/ml (**A**) and Ps#12 (**B**), which showed high amount of noscapine into collected ecotypes, are illustrated. The absorption is given in: absorption units (**AU**) and milli absorption units (**mAU**).

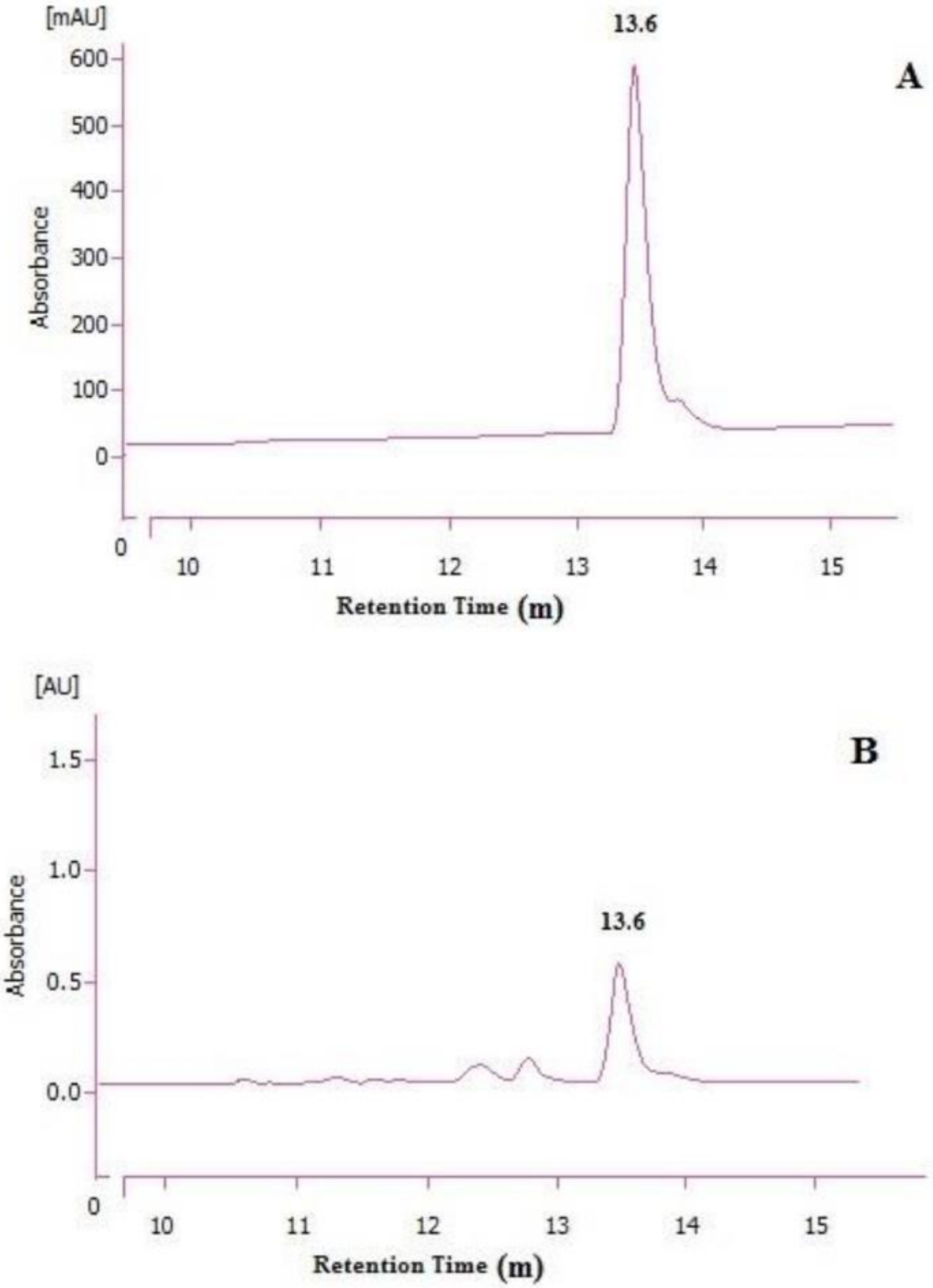

[mAU]
13.6
A
Absorbance
600
500
400
300
200
100
0
0
10
11
12
13
14
15
Retention Time (m)
[AU]
B
1.5
1.0
0.5
0.0
Absorbance
13.6
0
10
11
12
13
14
15
Retention Time (m)

Supplementary Information 10

S11. The full-length of gel pictures sued in the article

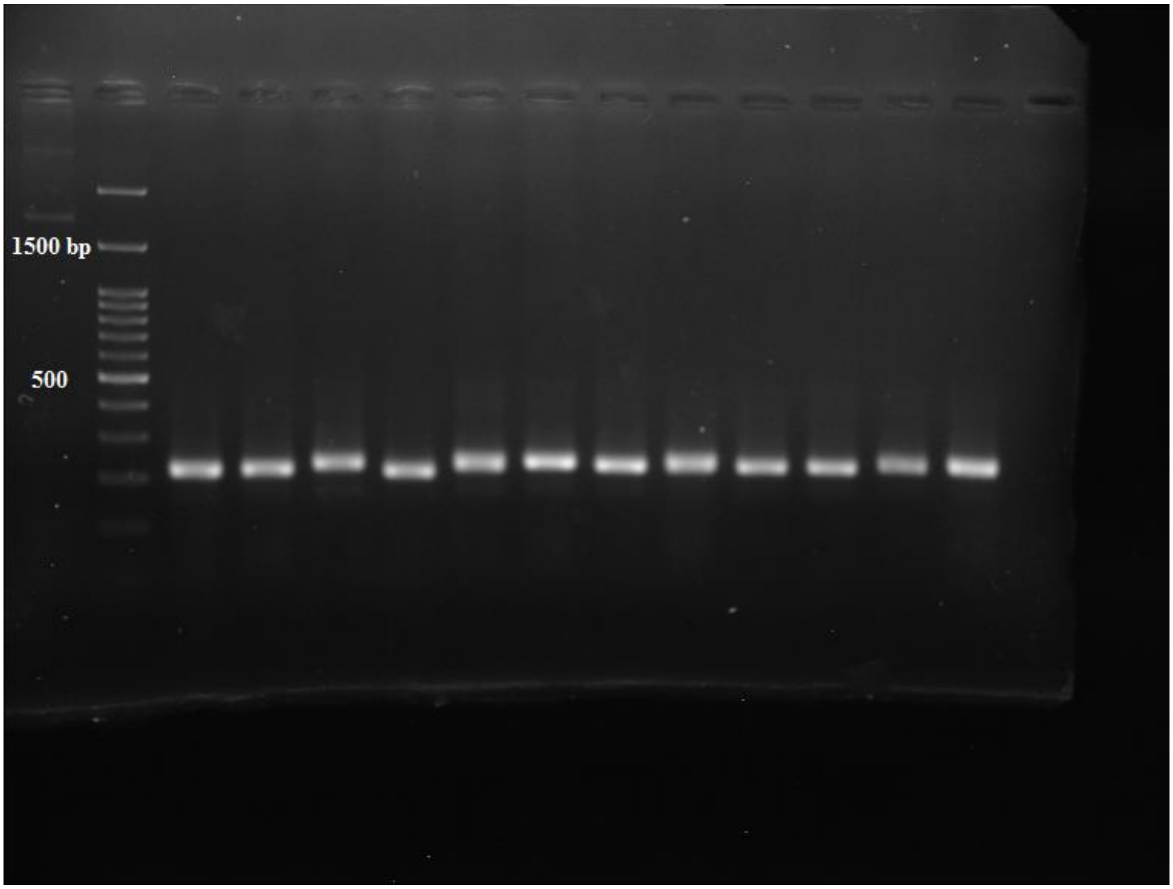

Full-length gel of Fig. 1. a

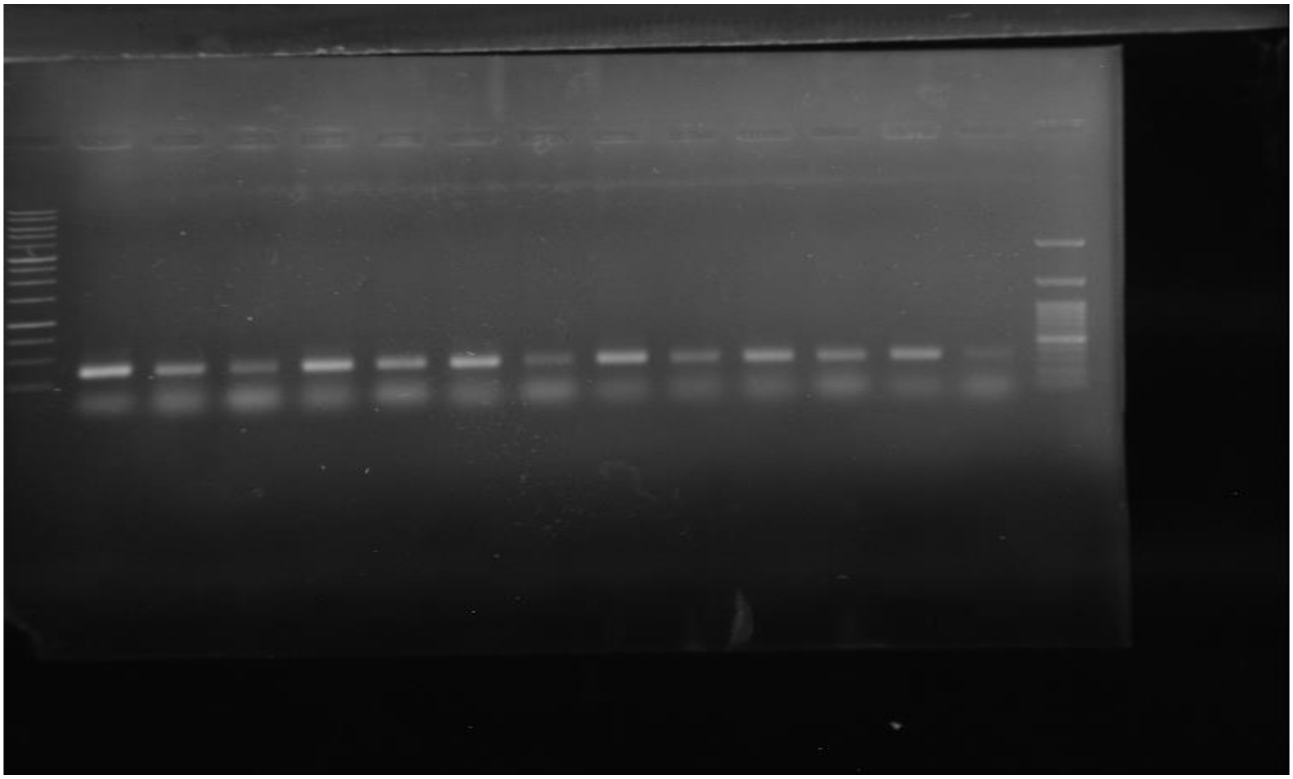

Full-length gel of Fig. 2
